# Supplementary material for: Intrinsic luminescence blinking from plasmonic nanojunctions
Source: Nat Commun. 2021 May 21;12:2731. doi: 10.1038/s41467-021-22679-y (PMC8139969; doi:10.1038/s41467-021-22679-y)
Supplement: Supplementary file 1 — Supplementary Information [file 41467_2021_22679_MOESM1_ESM.pdf]

Supplementary Material for Intrinsic Luminescence Blinking from Plasmonic Nanojunctions

Wen Chen[1], Philippe Roelli[1,2], Aqeel Ahmed[1], Sachin Verlekar[1], Huatian Hu[3], Karla Banjac[4], Magalí Lingenfelder[4], Tobias J. Kippenberg[2], Giulia Tagliabue[5], Christophe Galland[1*]

[1]Ecole Polytechnique Fédérale de Lausanne,
Laboratory of Quantum and Nano-Optics, Lausanne 1015, Switzerland
[2]Ecole Polytechnique Fédérale de Lausanne,
Laboratory of Photonics and Quantum Measurements, Lausanne 1015, Switzerland
[3]The Institute for Advanced Studies, Wuhan University, Wuhan 430072, China
[4]Ecole Polytechnique Fédérale de Lausanne,
Max Planck-EPFL Laboratory for Molecular Nanoscience, Lausanne 1015, Switzerland
[5]Ecole Polytechnique Fédérale de Lausanne,
Laboratory of Nanoscience for Energy Technologies, Lausanne 1015, Switzerland
*To whom correspondence should be addressed; E-mail: chris.galland@epfl.ch

Supplementary Methods

Sample fabrication and characterisation

Twenty-one types of plasmonic nanojunctions (see the list in Supplementary Table 1) were fabricated by a 4-step process: (1) fabrication of a metal film, (2) deposition of one or multiple layers on the metal film as a spacer, (3) drop-casting of metal nanoparticles on top of the spacer, and (4) growth of a compact Al_2O_3 layer on the sample surface for protection, the details of which are given in the following subsections.

Metal film fabrication

Four types of metal films were prepared: evaporated Au and Cr (AuCr) film, template-stripped gold (TSAu) and silver (TSAg) films, colloidal Au microplates (AuMPs). The AuCr film with root-mean-square (RMS) roughness of ∼1.6 nm[1] was fabricated by evaporating 5-nm-thick Cr film on a Si wafer, followed by 100-nm-thick Au layer with growth rate of 0.5 nm s^{-1}. To fabricate the TSAu film, 200-nm-thick gold film was firstly evaporated on a clean Si wafer using electron beam evaporation at the same deposition rate. Next, the Au surface was glued with pieces of ∼1 cm^2 glass using an optical adhesive (NOA61), cured by ultraviolet light. Then the Si and glass slices were peeled off by a razor, leaving fresh ultrasmooth Au surface on the glass substrate. The TSAg films were fabricated in the same way. Atomic force microscopy was used to characterise the surface roughness and crystal grain size distribution of template-stripped (TSAu) and as-evaporated AuCr films, shown in Supplementary Fig. 2. AFM images were collected by Dimension Fast Scan AFM (Bruker) coupled with Nanoscope V Controller (Bruker). FastScanB probes (Bruker) were used. All images were recorded in the air. Grain size analysis was conducted using Gwyddion software.

AuMPs were synthesized according to the method reported in Ref 2. Briefly, 6 mL ethylene glycol (Sigma-Aldrich) was firstly added into a 100 mL flask under 150 °C oil bath. Then 1 mL of 0.2 M gold(III) chloride hydrate ($HAuCl_4$, Sigma-Aldrich) aqueous solution was injected into the flask. Next, 3 mL ethylene glycol solution containing 0.666 g of dissolved polyvinylpyrrolidone (PVP, Mw = 40000, Sigma-Aldrich) was dropped into the flask. After 10 min, AuMPs start to appear in the solution. The reaction was terminated after another 20 min, and the AuMPs were cleaned by acetone and ethanol solution, and finally stored in ethanol solution. The average lateral size and thickness are ∼40 μm and 100 nm, respectively.

Spacer fabrication

Different nanomaterials, including self-assembled monolayer (SAM) biphenyl-4-thiol (BPhT), monolayer MoS_2, Al_2O_3, as well as native ligands on the colloid crystals, and their combinations, were used as spacers for the plasmonic nanojunctions, with thicknesses varying from 0.7

nm to 3 nm (Supplementary Table 1). SAM of BPhT molecules on the metal films with approximately 1 nm average thickness were obtained by immersing a fresh metal film in a BPhT ethanol solution with different incubation conditions. For AuCr, TSAu and TSAg films, 1 mM BPhT ethanol solution was used at room temperature for 2 hours incubation. In the case of the AuMPs, their solution was firstly drop-casted on a clean Si wafer and dried with nitrogen gas. Then the AuMP sample was immersed in a high concentration (0.1 M) BPhT solution at 70°C for 24 hours.

After the incubation the samples were all rinsed by 5 successive flows of ethanol and water to remove the extra BPhT molecules, and dried by nitrogen gas.

On some samples, 1-nm- or 1.5-nm-thick Al_2O_3 spacers were deposited either on the bare metal, on top of the BPhT SAM or the PVP-capped on AuMP. We used atomic layer deposition (ALD) at 100 °C. The growth process shows the negligible impact on the Raman signal of the BPhT (Figure S3), confirming that this temperature is low enough not to damage the molecules and SAM in a significant way. For the fabrication of a monolayer MoS_2 spacer, thin bulk MoS_2 was firstly transferred on a metal film by mechanical exfoliation method.[2] The sample was then annealed at 200°C for 8 hours, forming strong and uniform Au-MoS_2 bonding that also restructures the metal surface.[1] Next, the sample was immersed into acetone solution under ultrasound condition for 3 min to peel off the bulk MoS_2, leaving the bottom monolayer (or few-layer) MoS_2 on the metal surface.

Nanoparticle synthesis and their surface functionalisation

The AgNCs were synthesized by the protocol from Ref.[3] Briefly, H_2O solution of 5 mL 0.02 M CTAC (25 wt% in H_2O, Sigma-Aldrich) and 0.5 mL 0.1 M ascorbic acid (Sigma-Aldrich) were mixed in the glass vial for 10 min preheating at 60 °C. Then aqueous solutions of CF_3COOAg (50 µL, 10 mM) and $FeCl_3$ (80 µL, 4.3 µM) were added to the glass vial. After 3 hours reaction, the products were centrifuged at 14500 rpm and finally stored in aqueous solutions of 0.02 M CTAC.

To fabricate BPhT-covered AuNPs, 200 µL aqueous solution of AuNPs (BBI solutions) with original concentration (optical density 0.88 at 520 nm) were mixed with 200 µL aqueous solution of sodium citrate tribasic dihydrate (10 mM) and 600 µL ethanol solution of BPhT (10 mM) for the replacement. After 2 hours incubation, the products were centrifuged at 14500 rpm and finally stored in H_2O.

Nanoparticle drop-casting and ALD sealing

Plasmonic nanojunctions were formed by drop-casting AuNP solution on various spacer-film systems, where the coverage of the nanoparticles depends on the colloid concentration and the surface condition (hydrophobic vs. hydrophilic). After 30 s to 5 min incubation (depending on solution concentration), the samples were gently rinsed by water and dried by nitrogen gas. For AgNCs, the sample was dried after drop-casting, and then immersed into ethanol and H_2O to remove the residual CTAC molecules on the AgNCs. The CTAC and PVP molecules capped on the NPs and AuMP bring additional 1-2 nm spacer distance in the plasmonic nanocavities,[1]

and ∼0.5 nm thickness for citrate capped nanoparticles. For BPhT SAM and MoS_2 directly contacting with the ligand layer, the additional gap thickness would be further reduced due to the BPhT replacement and MoS_2-induced metal surface migration.[4,5] Finally, a 4 to 10 nm compact alumina layer was grown on the sample surface by ALD at 100°C. This oxide layer protection improves the long-term stability against oxidation and laser irradiation. As shown in Supplementary Fig. 3, the growth of Al_2O_3 on the sample surface results in the redshift of the plasmonic resonance, due to the combination of the increased charge screening effect and the slightly increased bottom facet size of the nanoparticles under heating during ALD.[4]

Spectroscopy

Simultaneous PL and Raman measurements

The simultaneous PL and Raman measurements with 532 nm and 750 nm excitation beams were implemented by the optical setup shown in Supplementary Fig. 4. Linearly polarised 750 nm continuous wave (cw) light from a Ti:Sa laser was sequentially directed to a noise-eater, a cleanup filter and a radial polarisation converter to form a clean and narrow 750 nm laser line with radial polarisation. The laser was eventually focused by a high numerical aperture (NA) objective to form a diffraction-limited laser beam on the sample, providing a large out-of-plane electric field component to effectively excite the gap mode of the nanojunctions. On the other hand, linearly polarised 532 nm cw light from a diode laser was directed and collimated though a fiber coupling system, and then pass through a cleanup filter, a radial polarisation converter and the same objective to form a radially polarised 532 nm laser beam with a clean and narrow spectral line on the sample. The sample was mounted on an 3-axis piezo-stage with displacement precision better than 100 nm, allowing for three-dimensional alignment of the single nanojunction with respect to the laser beam. The light from the sample was collected by the same objective, then passed through a group of short and long pass filters and a 532 nm notch filter to eliminate 532 nm and 750 nm light, eventually directed to the slit of a spectrometer. A part of the reflected light was directed to a camera to find the position of the nanoparticles by white light illumination and confirm the alignment of the 532 nm and 750 nm laser beams. All the raw spectra were subtracted by noise spectra with the same integration time.

Simultaneous PL and DF measurements

The PL and/or dark-field (DF) measurements were implemented by the optical setups shown in Supplementary Fig. 5. For DF measurements, white light from a halogen lamp was guided by a multimode fiber, collimated and refocused by two lenses placed on the side of the sample stage, with the beam making an angle of 10° with the plane of the sample. A polariser was placed in between the lenses to convert unpolarised white light to linearly polarised light, enabling p- or s-polarisation excitation. The scattered light from the sample (without specular reflection) was collected by an objective lens and directed to a camera for DF imaging or coupled to a multimode fiber connecting to a spectrometer for DF spectroscopy. The 0.2 mm core size of the fiber gives a circular collection area with a diameter $\sim 2\,\mu\mathrm{m}$ on the sample, allowing for the scattered signal from only one single nanojunction to enter the spectrometer. A background

spectrum was acquired from the bare metal film near the measured nanojunction. The net DF spectrum was then obtained by background subtraction and then divided by the spectrum acquired by directing the white light into the objective (using an angled micro-mirror). This calibration accounts for both the intrinsic lamp spectrum and the setup spectra response. The sample was mounted on a 3-axis piezo-stage with displacement precision better than $\sim$100 nm, allowing for precise alignment between the nanoparticle and the collection area.

For simultaneous PL and DF measurements, a 532 nm laser beam was directed to the objective after a clean-up filter, forming a highly focused beam adjusted to the center region of the collection area. The PL signal was collected in the same way as the DF signal, after blocking wavelengths shorter than 550 nm with a dichroic mirror and a long pass filter. A 532 nm notch filter was placed in front of the camera to image the PL. The PL measurements implemented with the setup in Supplementary Fig. 5b follows the same laser excitation and signal collection paths. A dark-noise spectrum acquired with the same integration time was subtracted from each raw spectrum.

Temperature dependent PL and/or Raman measurements

The variable-temperature PL and/or Raman measurements were implemented by a cryogenic system shown in Supplementary Fig. 6. Before the optical measurement, the sample was glued on a 3-axis piezostage (attocube) integrated inside the cryostat by elargol. The vacuum of the chamber was pumped down to $< 5\times10^{-6}$ mbar, following modifby cooling down to a base temperature of 3.8 K. The control of the sample temperature above 3.8 K was realized by a heating system integrated on the sample holder, with the precision of 0.1 K. For spectroscopy, linearly polarised beams from a Ti:Sa laser tuned to 740 nm, and two other fixed frequency lasers (diode laser at 532 nm and HeNe laser at 633 nm) were overlapped with the help of dichroic mirrors, and reflected on a beam-splitter into the objective. A single nanojunction was aligned to the focus position by a piezo-stage with three-dimensional displacement precision better than 10 nm. The output light from the sample was collected by the same objective, passed through tunable bandpass filters and notch filters to eliminate 532, 633 nm and 740 nm light. The signal was then either focused into the slit of a spectrometer or into a fiber-coupled to a single photon counting avalanche photodiode (APD) to measure high-speed intensity traces. Part of the reflected light was directed to a camera to confirm the alignment of the laser beams and to find the position of the nanojunctions under white light illumination. A dark-noise spectrum acquired with the same integration time was subtracted from each raw spectrum.

Simulation

The full-wave simulations were performed on a commercial software package (COMSOL Multiphysics 5.2a). An 80-nm-diameter Au nanoparticle with 40-nm-diameter bottom facet size (truncated sphere) was separated from the Au film by a 1.3- nm-thick layer (refractive index = 1.4). The dielectric function of the gold followed experimental data from Johnson & Christy.[6] For the simulation shown in the main text, oscillating electric point dipoles were placed on the Au film (the position from symmetry axis: x = 0 nm, 12 nm, 17 nm) to mimic the PL radiation

from localised emitters. The local enhancement of the photonic density of states vs. frequency was calculated by comparing the dipole radiation with and without the plasmonic geometry. A collection cone was applied in the integration to keep only the emission inside a 0.85 numerical aperture. In the experiment, we propose that the randomness of the emitter position makes it couples randomly to different modes. Plane-wave excitation was also applied to calculate the basic scattering/absorption spectra (the main text), and clarify the plasmon field and charge distribution.

For the simulation in Supplementary Fig. 12, a 150-nm-diameter AuNP with 70 nm facet is placed on the silica (refractive index = 1.5) substrate. A 1- nm-thick gap is set between the NP and substrate due to represent the ligand. The scattering is calculated by illuminating a plane wave with an 80 degree incident angle from the surface.

To investigate the laser-induced thermal effect, a 532 nm laser beam with $\sim 3.5 \times 10^4$ W/cm^2 intensity illuminated the nanojunction. The electromagnetic simulation was performed to derive the heat absorption in the metal, which is then plugged into the heat transfer model as a heat source. The thermal conductivity of the Au and spacer (ALD, k_{Gap}) was set as 314 W/(m·K) and 1.8 W/(m·K), respectively, while the heat capacity of the two materials was set as 129 J/(kg·K) and 755 J/(kg·K).[7,8]

No.	Name	Nanoparticle	Spacer	Mirror	Al_2O_3 coating
**1**	AuNP-BPhT-TSAu	80-nm P-AuNP	BPhT	200-nm-thick TSAu (the same below)	4 nm
**2**	AuNP-BPhT-TSAu	80 nm R-AuNP	BPhT	TSAu	0-8 nm
**3**	AuNP-BPhT-AuMP	80 nm P-AuNP	BPhT	~100-nm-thick AuMP (the same below)	5-10 nm
**4**	AuNP-BPhT-AuCr	80 nm R-AuNP	BPhT	100 nm Aufilm + 5 nm Crfilm	0 nm
**5**	AgNC-BPhT-TSAu	60 nm AgNC	CTAC (on AgNC) + BPhT (on mirror)	TSAu	5 nm
**6**	AgNC-BPhT-AuMP	60 nm AgNC	CTAC (on AgNC) + BPhT (on mirror)	AuMP	5-10 nm
**7**	AuNP-Al_2O_3-BPhT-AuMP	80 nm P-AuNP	Al_2O_3 + BPhT	AuMP	5 nm
**8**	AuNP-Al_2O_3-BPhT-TSAu	80 nm P-AuNP	Al_2O_3 + BPhT	TSAu	5 nm
**9**	AgNC-Al_2O_3-BPhT-AuMP	60 nm AgNC	CTAC (on AgNC) + 1-nm-thick Al_2O_3 + BPhT (on mirror)	AuMP	5 nm
**10**	AuNP-PVP-AuMP	80 nm P-AuNP	PVP (on mirror)	AuMP	0-5 nm
**11**	AuNP-Al_2O_3-PVP-AuMP	80 nm P-AuNP	1-nm-thick Al_2O_3 + PVP (on mirror)	AuMP	0-5 nm
**12**	AuNP-MoS_2-TSAu	80 nm R-AuNP	1L MoS_2	TSAu	0-8 nm
**13**	AgNC-MoS_2-TSAu	60 nm AgNC	1L MoS_2	TSAu	0-8 nm
**14**	AgNC-Al_2O_3-MoS_2-TSAu	60 nm AgNC	1-nm-thick Al_2O_3 + 1L MoS_2	TSAu	0-8 nm
**15**	SiO_2-AuNP-BPhT-TSAu	2-nm-thick SiO_2 covered 80 nm AuNP	2-nm-thick SiO_2 + BPhT	TSAu	0-8 nm
**16**	SiO_2-AuNP-TSAu	80 nm SiO_2-AuNP	2-nm-thick SiO_2	TSAu	0-8 nm
**17**	AuNP-Al_2O_3-TSAu	80 nm P-AuNP	1-nm-thick Al_2O_3	TSAu	0-8 nm
**18**	AgNC-CTAC-Al_2O_3-TSAg	60 nm AgNC	CTAC (on AgNC) + 1.5-nm-thick Al_2O_3 (on mirror)	200-nm-thick TSAg	0-8 nm
**19**	AuNP-TSAu	80 nm R-AuNP	Citrate	TSAu	0-8 nm
**20**	BPhT-AuNP-TSAu	BPhT-capped P-AuNP	BPhT (on AuNP)	TSAu	0-8 nm
**21**	BPhT-AuNP- Al_2O_3-TSAu	BPhT-capped P-AuNP	BPhT (on AuNP) + 1.5-nm-thick Al_2O_3 (on mirror)	TSAu	0-8 nm

Supplementary Table 1: List of plasmonic nanojunctions investigated. P-AuNP and R-AuNP (see Supplementary Fig. 1a): polyhedral and rounded Au nanoparticle, AuMP: Au microplate, AgNC (see Supplementary Fig. 1b): Ag nanocube, TSAu and TSAg: template-stripped Au See Supplementary Fig. 2a) and Ag film.

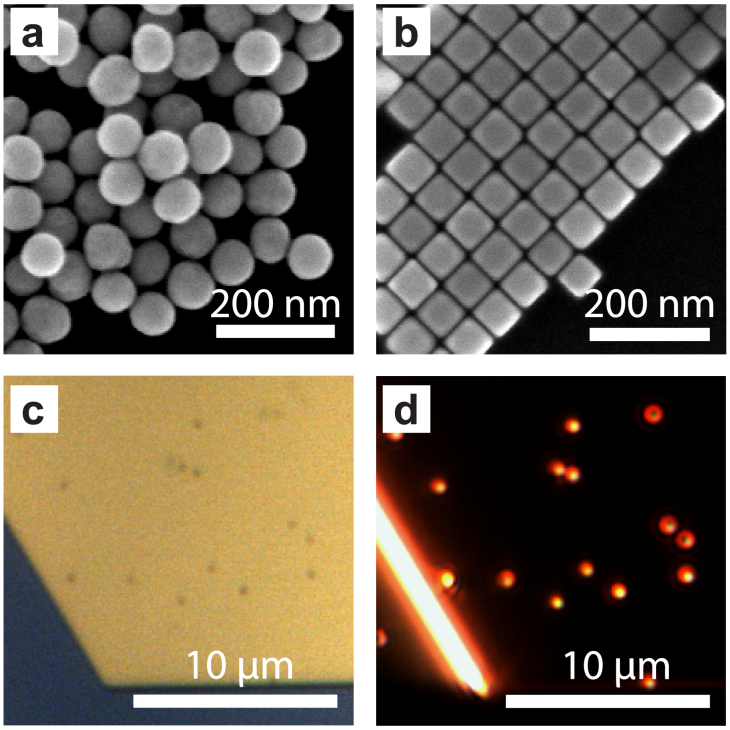

Supplementary Figure 1: SEM and optical image of nanoparticles and nanojunctions. (**a, b**) SEM top view of (**a**) gold nanospheres with ∼80 nm diameter (Expedeon) and (**b**) silver nanocubes with ∼ 60 nm edge size (home made). (**c**) Top view of bright field and (**d**) DF scattering images of AuNP-BPhT-AuMP nanojunctions (No.3 in Supplementary Table 1). Nanoparticles can be well located even under bright field illumination as darker spots.

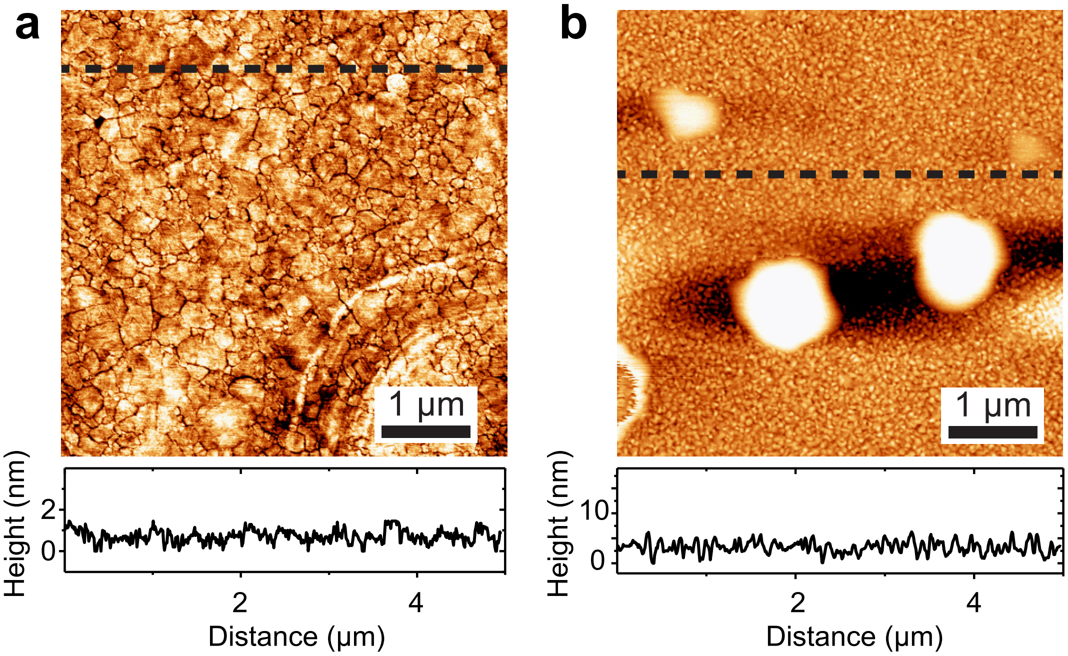

Supplementary Figure 2: AFM images of Au film surfaces. (**a, b**) Representative atomic force microscope (AFM) images of TSAu (**a**) and AuCr (**b**) films, with height profiles along the lines superimposed on each image shown in the bottom panel. Based on grain size analysis we find that the mean grain size of TSAu and AuCr are 154±19 nm and 45±21 nm, respectively. Bright and irregular features on AuCr film (**b**) are most likely contaminants.

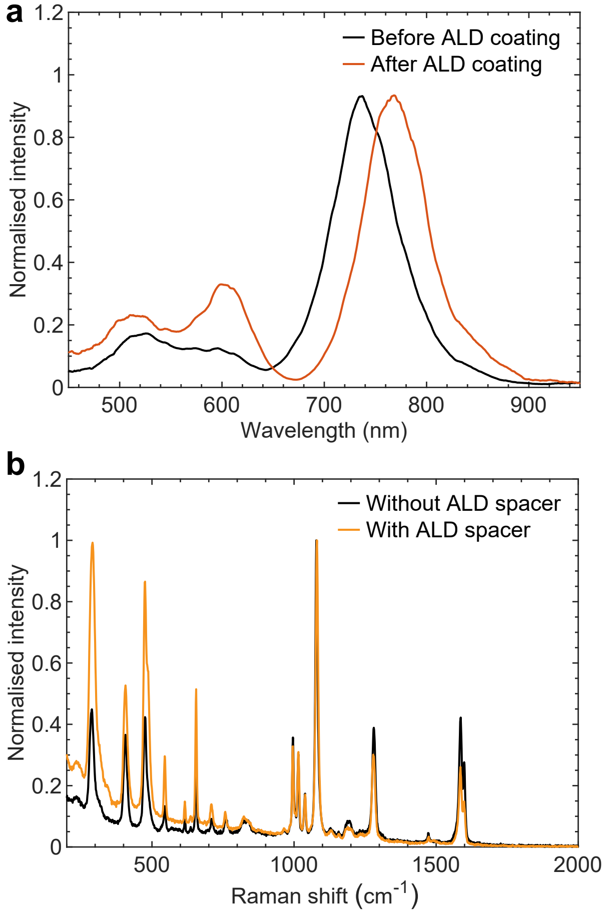

Supplementary Figure 3: Impact of ALD used for spacer or capping layer. (**a**) DF scattering spectra of a plasmonic nanojunction before and after Al_2O_3 capping of the sample. The redshift is expected due to the increase in the dielectric constant around the particle. (**b**) Raman spectra of the BPhT plasmonic nanojunction with (No. 3 in Supplementary Table 1) and without (No. 7 in Supplementary Table 1) Al_2O_3 layer as an additional spacer. The slightly increased Raman signal at small Raman shift relative to large Raman shift when the additional ALD spacer is inserted may be due to a shift of the plasmonic resonances (blue shift because of a larger gap) closer to the laser wavelength of 785 nm in this case.

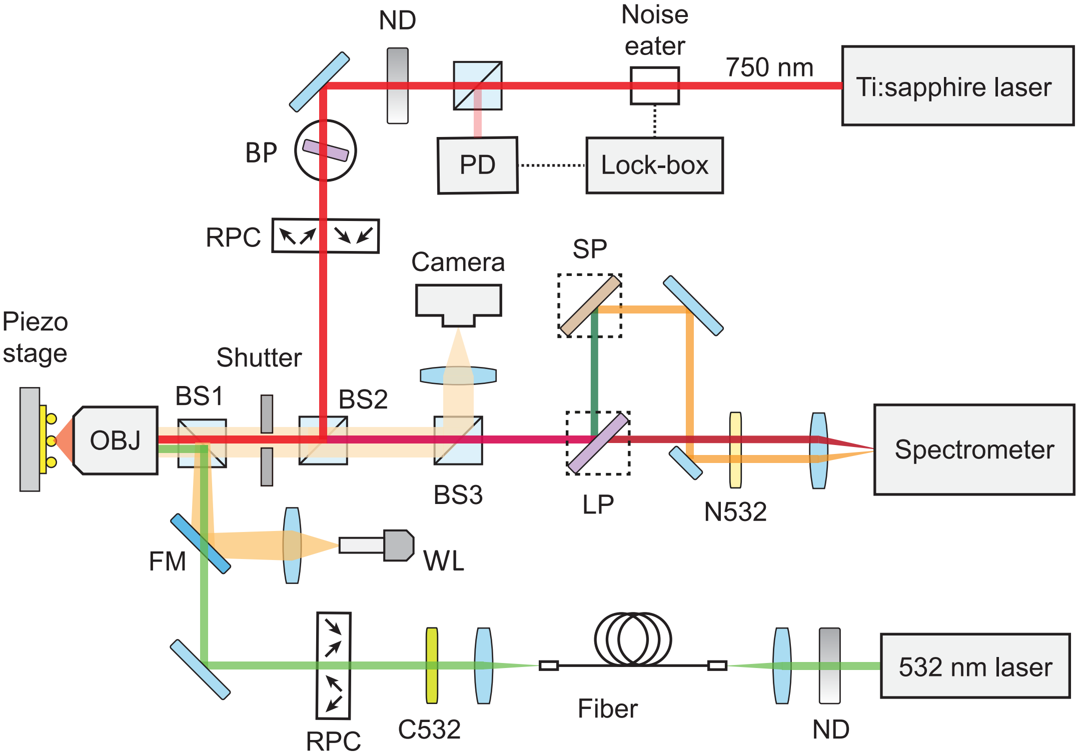

Supplementary Figure 4: Schematic of the light path for simultaneous PL and Raman measurement. ND: neutral density filter, FM: flip mirror, BP: tunable bandpass filter, RPC: radial polarisation converters, PD: photodetector, WL: white light source, BS1: beamsplitter with reflection:transmission in % (R:T) = 10:90, BS2: beamsplitter with R:T = 20:80, BS3: flip pellicle beamsplitter with R:T = 8:92, LP: AHF tunable longpass filter module, SP: AHF tunable shortpass filter module, N532: notch filter centered at 532 nm, C532: cleanup filter centered at 532 nm, OBJ: Nikon objective, numerical aperture = 0.95, working distance = 0.21 mm, Spectrometer: Andor Shamrock, grating: 300 l/mm, CCD: Andor iDus 416, Tunable Ti:Sa laser: Coherent MBR, tuned to 750 nm. 3-axis stage: The sample is mounted on an 3-axis piezo stage with displacement precision better than 100 nm.

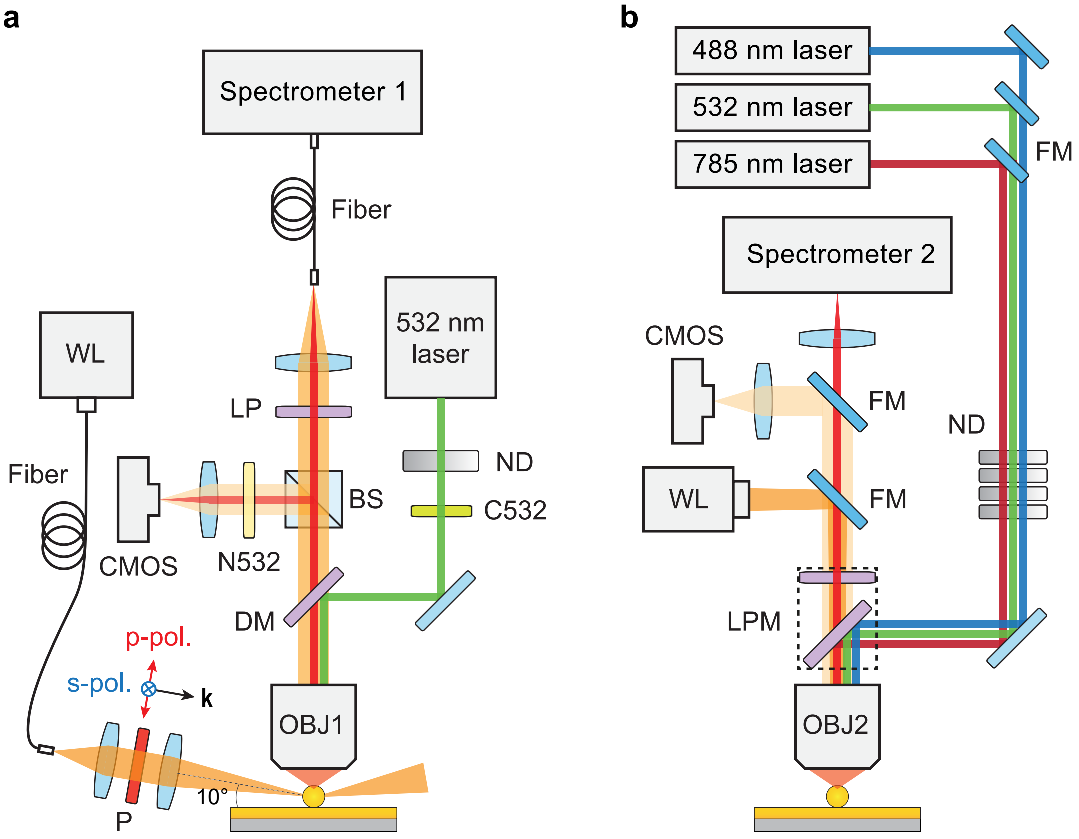

Supplementary Figure 5: Schematic of the optical setups for PL and dark-field (DF) spectroscopy (**a**) ND: neutral density filter. C532: cleanup filter at 532 nm. P: broadband polariser. DM: dichroic mirror. N532: notch filter centered at 532 nm. LP: 540 nm longpass filter. BS: beamsplitter with R:T = 30%:70%. FM: beamsplitter with R:T = 10%:90%. WL: white light source. OBJ1: objective (Olympus), numerical aperture = 0.8, working distance = 3.1 mm. Spectrometer1: QEpro, Ocean Optics with a grating of 100 lines/mm, coupled via a 0.1 mm core size multimode fiber. (**b**) Spectrometer2: Renishaw inVia, 300 l/mm for 488 nm and 532 nm laser and 1200 l/mm grating for 785 nm laser. LPM: switchable longpass filter module. FM: flip mirror. OBJ2: objective (Leica), numerical aperture = 0.85, working distance = 1 mm.

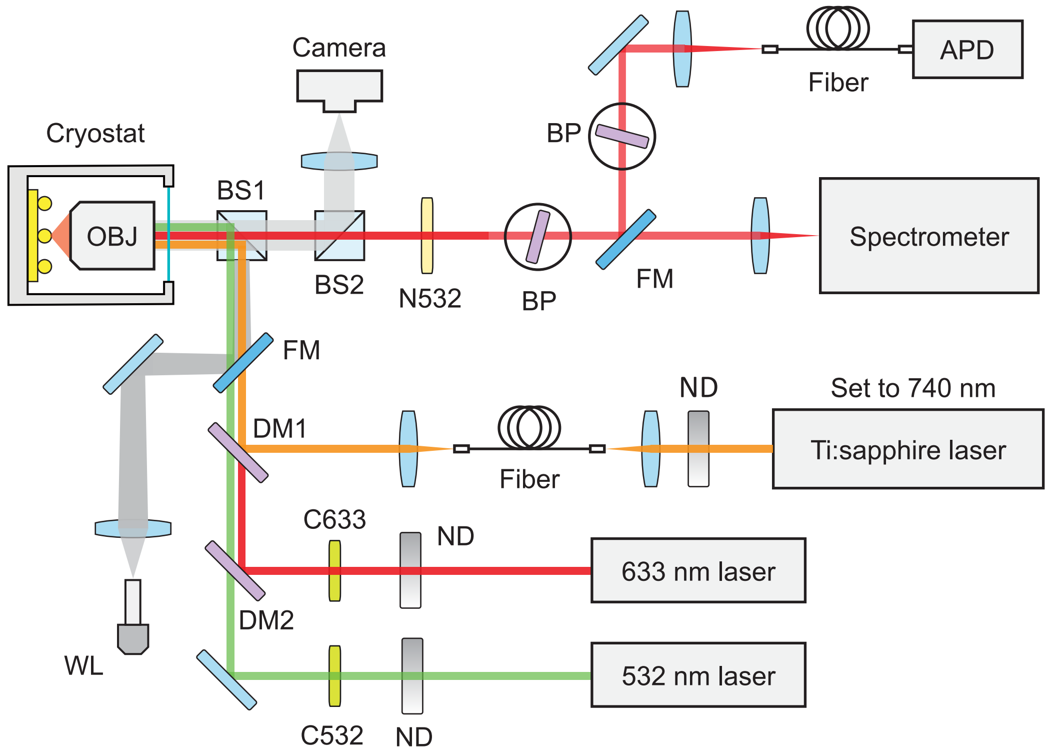

Supplementary Figure 6: Schematic of the light path for PL +Raman measurement at 3.8-300 K. ND: neutral density filter. BS1: beamsplitter R:T = 8%:92%. BS2: beamsplitter with R:T = 10%:90%. C532 and C633: cleanup filter at 532 nm and 633 nm. N532: notch filter at 532 nm. BP: tunable bandpass filter module covering 550-950 nm with bandwidth ∼100 nm. WL: supercontinuum source used for bright field imaging. FM: flip mirror. DM1: dichroic mirror. DM2: dichroic mirror. Ti:sapphire laser: Spectra-Physics 3900S, tuned to 740 nm. Cryostat: closed-cycle (cyostat attoDRY800, Attocube), the objective and piezo stage are integrated inside the chamber. OBJ: objective, numerical aperture = 0.81, working distance = 0.5 mm. Spectrometer: Andor Kymera 193i, grating: 600 l/mm. APD: a single-mode fiber-coupled avalanche photodiode, Excelitas Technologies.

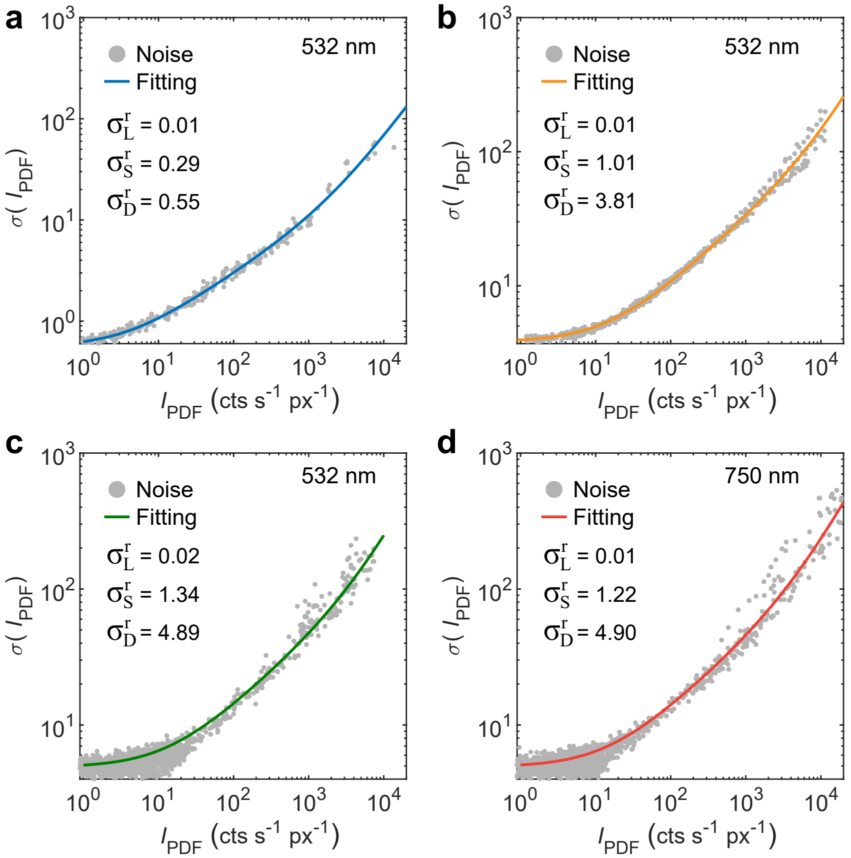

Supplementary Figure 7: Analysis of measurement noise. Measurement noise of the setup used (**a**) in Supplementary Fig. 5b and (**b**) in Supplementary Fig. 6 (**c** and **d**) in Supplementary Fig. 4, all of which were used to acquire the blinking traces reported in the main text. These data were acquired on the laser beam attenuated to obtain count rates matching the range of PL and/or Raman count rates typically observed. For a given mean signal intensity I the standard deviation of the intrinsic measurement noise $\sigma(I)$ has contributions from classical laser intensity fluctuations $\sigma_{\mathrm{L}} = \sigma_{\mathrm{L}}^{\mathrm{r}} I$, scaling linearly with I, from photon shot noise $\sigma_{\mathrm{S}} = \sigma_{\mathrm{S}}^{\mathrm{r}}\sqrt{I}$, and from detector noise σ_{D} (mainly readout noise of the CCD; for the short acquisition times used here modifthe dark current is negligible), according to $\sigma(I) = \sqrt{(\sigma_{\mathrm{L}}^{\mathrm{r}} I)^2 + (\sigma_{\mathrm{S}}^{\mathrm{r}}\sqrt{I})^2 + \sigma_{\mathrm{D}}{}^2}$.[9] This formula is used to fit the raw data, with the parameters shown in the figure. The shaded blue areas in the probability density function (PDF) in the main text and supplementary materials follow a Gaussian distribution given by $f_{\mathrm{PDF}}(I) = \exp\left[-(I - I_{\mathrm{PDF}})^2/2[\sigma(I_{\mathrm{PDF}})]^2\right]$, where I_{PDF} is the maximum of the PDF of the intensity trace to be calculated. In the time traces the blue areas are defined as $I_{\mathrm{PDF}} - 3\sigma(I_{\mathrm{PDF}}) \leq I \leq I_{\mathrm{PDF}} + 3\sigma(I_{\mathrm{PDF}})$, where I_{PDF} is used to present the average value of the baseline PL.

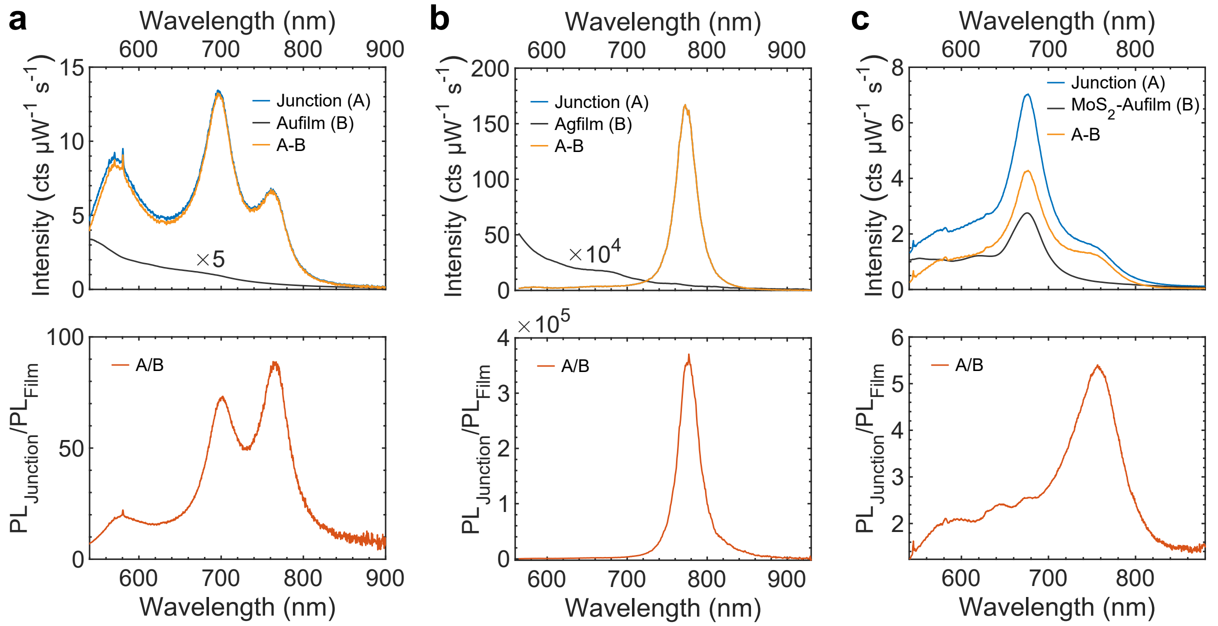

Supplementary Figure 8: Normalisation of PL spectra. (**a**) (top panel) Example of time-averaged PL spectrum when the objective focus is centered on a Au nanojunction (labeled A), or on an area of Au film without nanoparticle (magnified 5 times for visibility, labeled B). The PL spectra shown in this article are obtained by subtracting the latter from the former (A-B). The nanojunction PL enhancement factor can be estimated by dividing the nanojunction PL by the film PL: (A-B)/B$\simeq$A/B (the last approximation is valid because B$\ll$A). But this formula does not account for the much smaller area of the nanojunction compared with the spot size, and thus yields enhancement factors that are about 3 orders of magnitude underestimated.[10,11] (**b**) The corresponding spectra for an Ag nanojunction and the bare Ag film and their subtraction and division results. The film emission had to be magnified 10^4 times to be visible on the same scale. (**c**) PL spectra of a monolayer MoS_2 spaced Au nanojunction and the MoS_2 reference spectrum on a gold film (bottom panel). The excitonic emission of monolayer MoS_2 is largely quenched due to metal doping[4,12] but is still visible and stable. Using PL of the MoS_2 instead of the bare film spectrum as the reference enables us to extract the plasmonic response from the A-exciton background of MoS_2 (lower panel), which shows a metal-related PL peak at longer wavelengths. In this particular case, we cannot exclude that a dark exciton is rendered brighter by the Purcell effect and contributes to the new plasmonic emission.

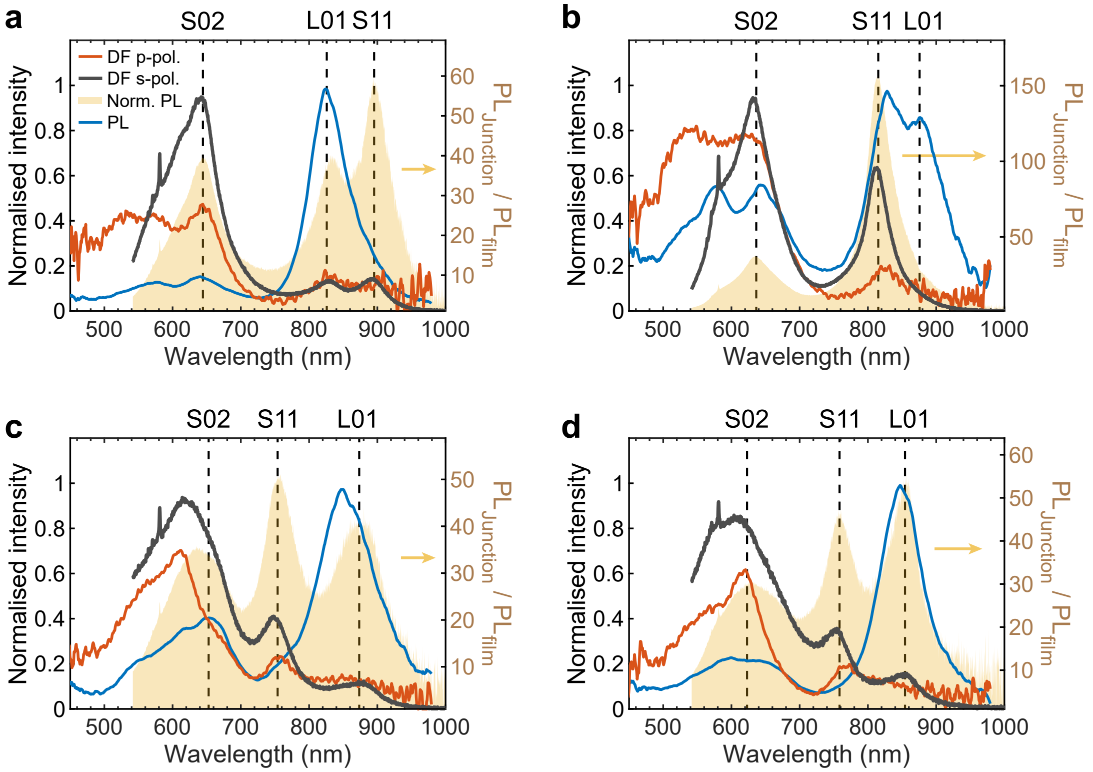

Supplementary Figure 9: Comparison of PL and polarisation-dependent DF spectra DF scattering spectra of 4 nanojunctions (No. 3 in Supplementary Table 1) excited by s- and p-polarised white light (see the detailed setting in Supplementary Fig. 5), along with their normalized time-averaged PL spectra under 532 nm excitation. Under p-polarised excitation, L01, S11 and S02 modes can all be excited, with scattering from the L01 antenna mode generally dominating the spectrum. With s-polarised excitation, scattering from S11 and S02 can still be detected with similar efficiency, while the L01 mode is much less visible, sometimes invisible. Our results agree well with our simulation analysis and previous reports.[4] Interestingly, the PL spectral profile typically matches better that of the s-polarised DF than that of p-polarised DF. In particular, the S11 mode seems to be excited more efficiently than the L01 mode. The origin of this feature could be associated with the better spatial overlap of the near field distribution of the S11 mode with that of the transverse mode (resonant with PL excitation, see Supplementary Fig. 20). After dividing PL_{Junction} by PL_{Film} (shaded areas) to eliminate the spectral variations related to the electronic structure of the metal, we find a qualitative match between the normalized time-averaged PL and the DF spectra.

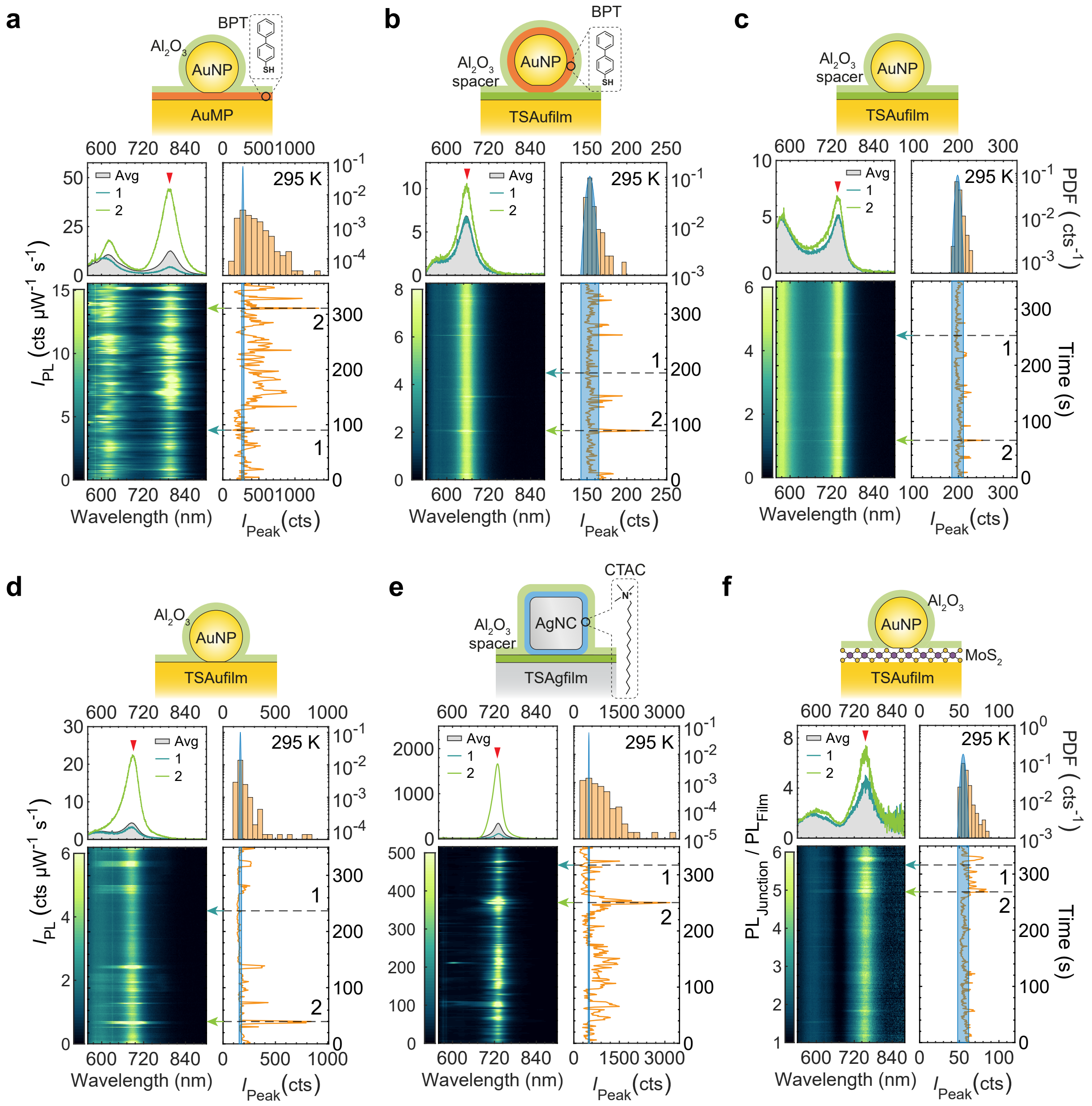

Supplementary Figure 10: Comparison of plasmon-enhanced PL blinking from different nanojunction assemblies. Representative time traces of plasmonic PL emission under continuous 532 nm excitation (full spectra as color plot and peak intensity as the orange line), with examples of single spectra (top-left panel) and probability density function (PDF) for the peak intensity (top-right panel). The blue shaded areas show the irreducible measurement noise for the same count rate. NP: nanoparticle; MP: micro-plates; BPhT: biphenyl thiol; TS: template-stripped; CTAC: cetyltrimethylammonium chloride. TSAufilm: template-stripping Au film. AuMP: Au micro-flake. Exposure time is 1s for all panels. The 532 nm laser was focused through a 0.85 NA objective with 37 μW incident power (power density $\sim 7.5 \times 10^3$ W cm^{-2}) – except for panel **a** (30 μW) and **f** (19 μW).

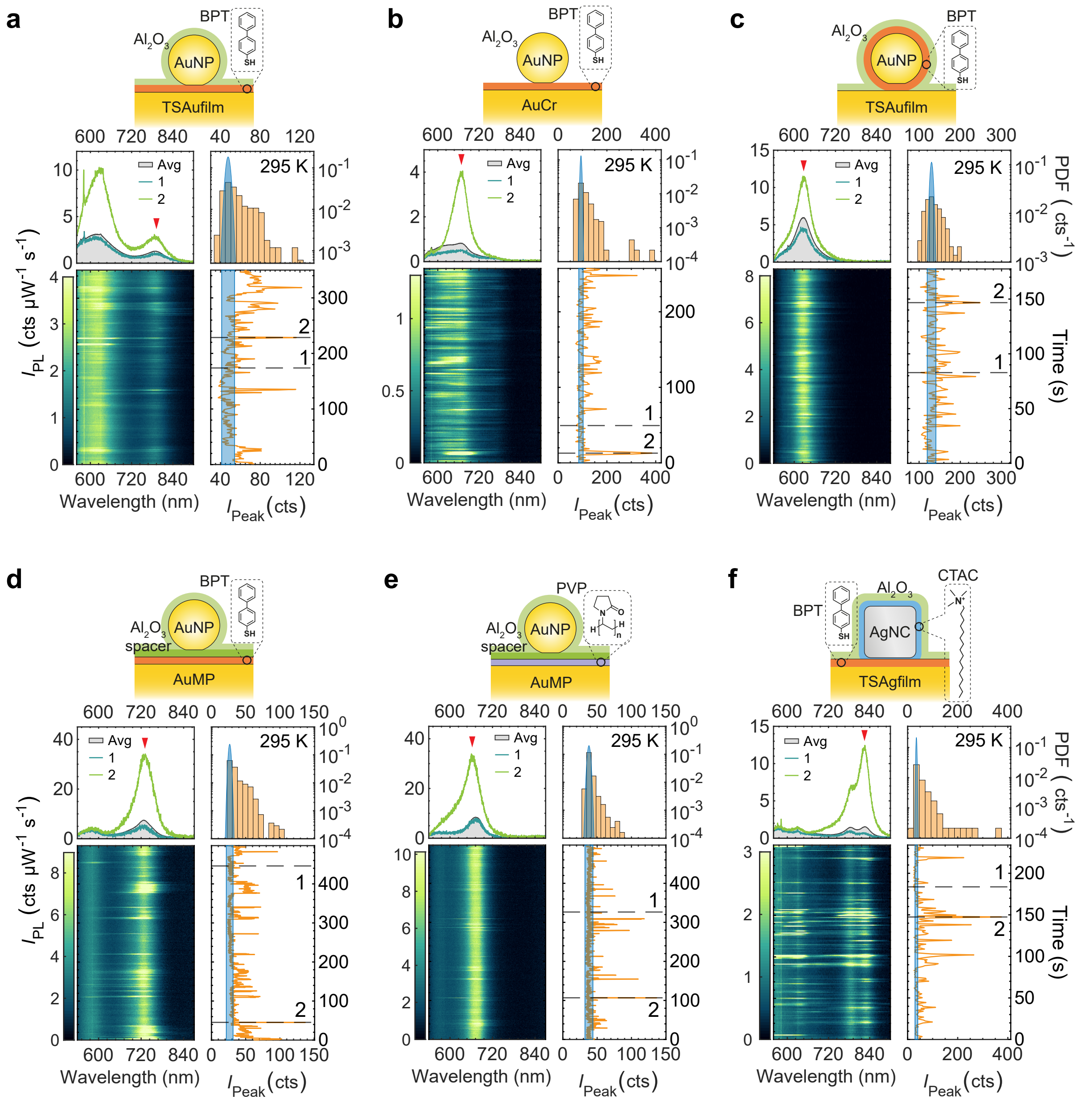

Supplementary Figure 11: More nanojunction examples of PL blinking. Time series of PL under 532 nm excitation at room temperature from (**a**) a AuNP-BPhT-TSAu nanojunction (from sample No. 1 in Supplementary Table 1), (**b**) a AuNP-BPhT-AuCr nanojunction (from sample No. 4), (**c**) a BPhT-AuNP-TSAu nanojunction (from sample No. 20), (**d**) a AuNP-Al_2O_3-BPhT-AuMP nanojunction (from sample No. 7), (**e**) a AuNP-Al_2O_3-PVP-AuMP nanojunction (from sample No. 11), and (**f**) AgNC-BPhT-TSAu nanojunction (from sample No. 5). The laser intensity was adapted to give well-resolved signal in each case, and remains in the range of few tens of microwatts or less.

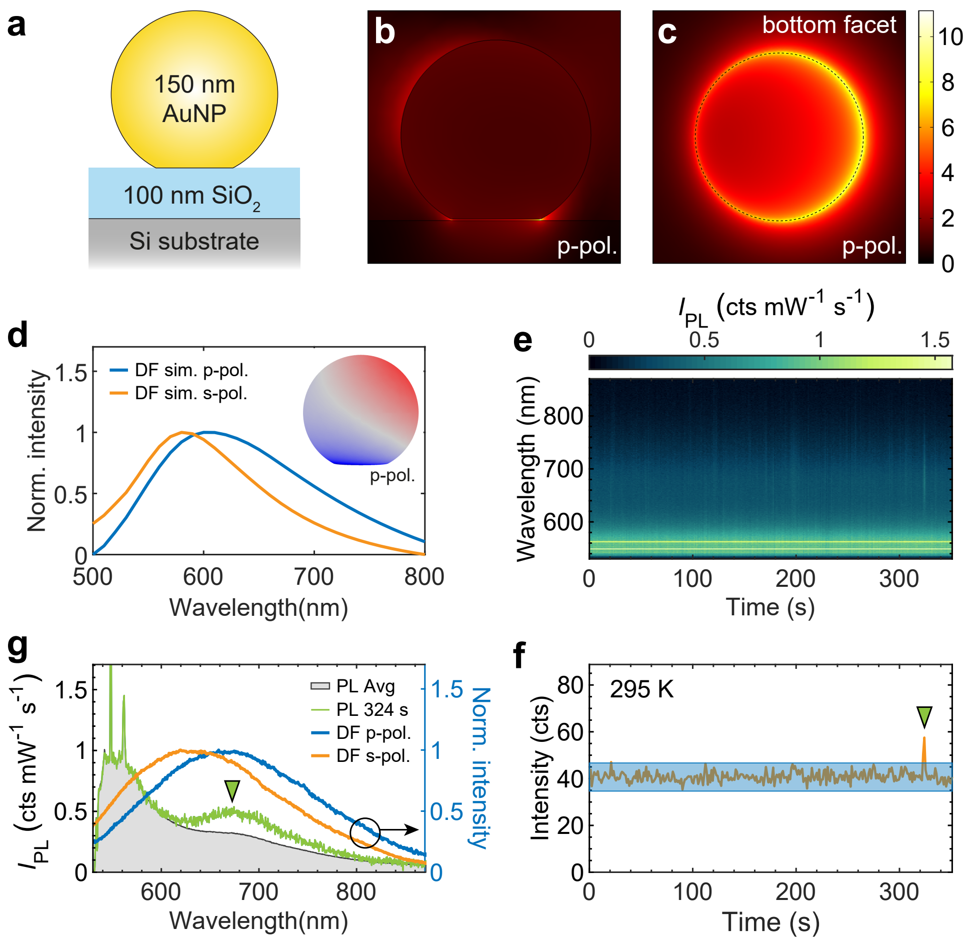

Supplementary Figure 12: Investigation of an individual 150-nm-diameter Au nanoparticle. (**a**) Schematic of a 150 nm Au nanoparticle on 100-nm-thick SiO_2 over a Si substrate. (**b**) Vertical cross-section and (**c**) in-plane bottom facet view of simulated near field enhancement under p-polarised excitation with a plane wave (k-vector making a 10° angle with substrate). Details of simulation settings are presented in Sec. . (**d**) Simulated far-field scattering spectra under p- and s-polarised (defined in Supplementary Fig. 5 and Fig. 1b in the main text) white light illumination. The inset shows the charge distribution at 600 nm under p-polarised excitation, identified as a substrate modified dipole mode. (**e**) An intensity map of PL time series from a Au nanoparticle under 532 nm excitation. Laser power, 110 µW; exposure time, 1s. (**f**) The intensity trace of the PL maximum at the dipole mode compared with the measurement noise. (**g**) Measured DF spectra under p- and s-polarised white light illumination, and PL spectra of the blinking event and of the time-average from (**e**). The two peaks around 550 nm are first- (saturated in the figure) and second-order Raman peaks of Si.

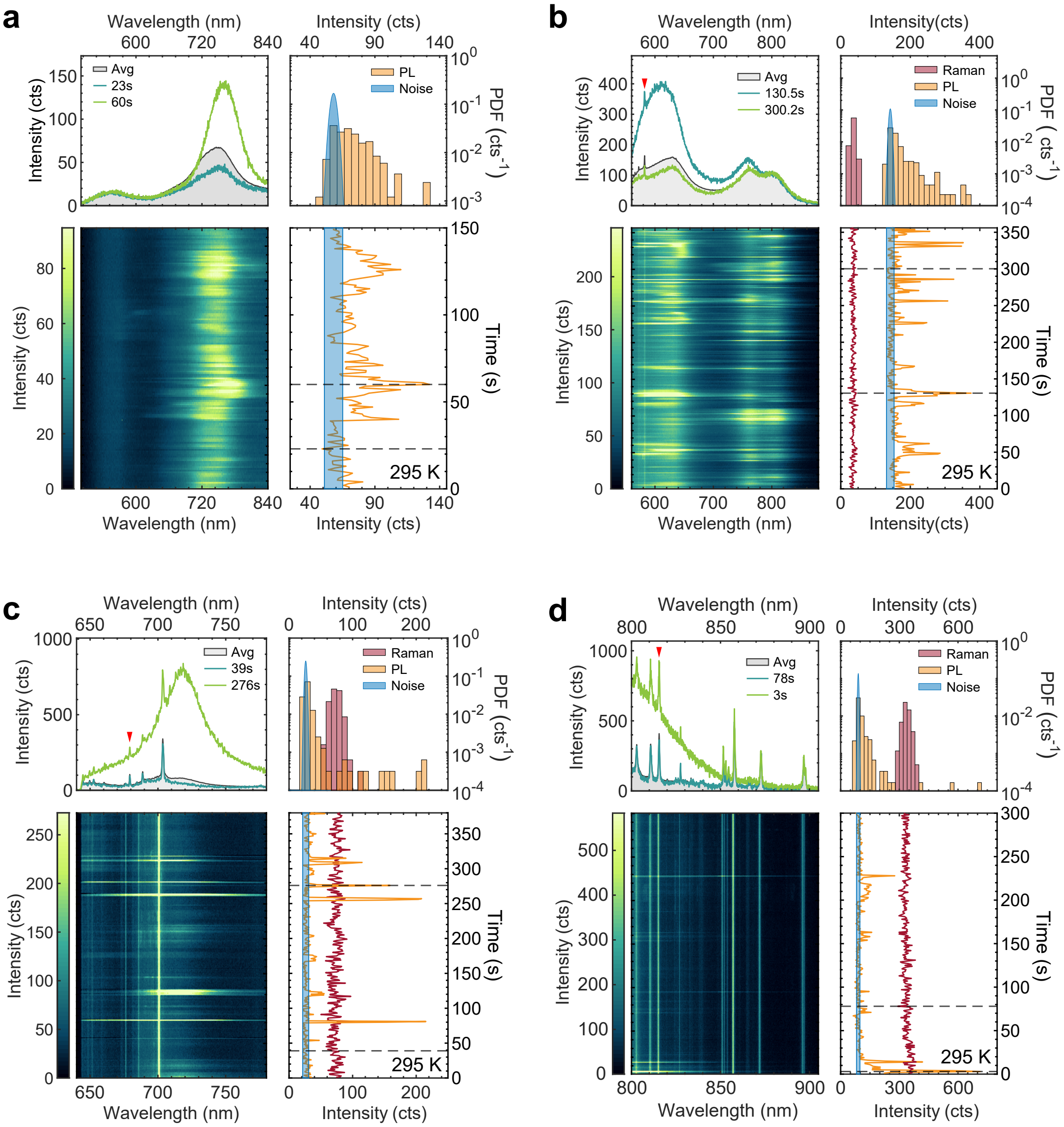

Supplementary Figure 13: Wavelength-dependent PL measurements. Time series of PL (and Raman) spectra from nanojunctions as a function of excitation wavelength. (**a**) Sample No. 3 in Supplementary Table 1 with 488 nm excitation with a power density of $\sim 1 \times 10^4$ W/cm^2. (**b**) Sample No. 3 with 532 nm excitation with a power density of $\sim 3 \times 10^3$ W/cm^2. . (**c**) Sample No. 18 with 633 nm excitation with a power density of $\sim 8 \times 10^3$ W/cm^2. (**d**) Sample No. 3 with 785 nm excitation with a power density of $\sim 2 \times 10^4$ W/cm^2. Note that under 785 nm excitation only the intraband transition and electronic Raman scattering are accessible. Exposure time are all 1 s.

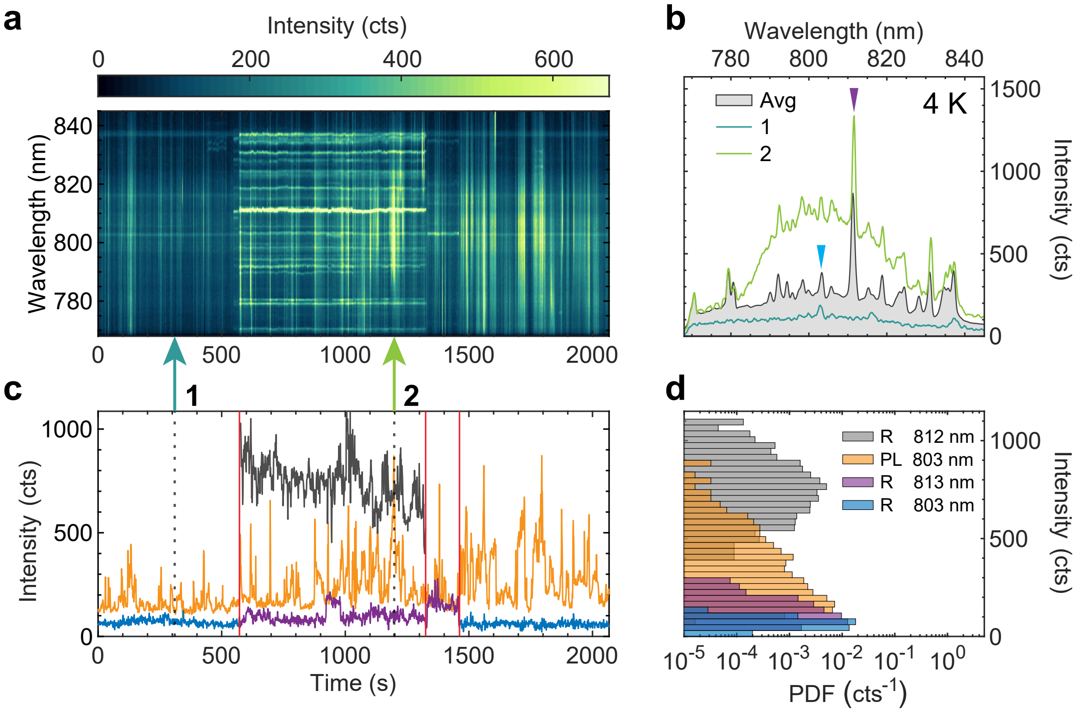

Supplementary Figure 14: Simultaneous PL and Raman measurement at cryogenic temperature. Different mechanisms for PL blinking and Raman fluctuations at 3.8 K (sample temperature) from sample No. 2 measured by the setup in Figure 6. (**a**) Time series (color map) and (**b**) representative individual spectra of the emission from a single nanojunction under simultaneous 532 nm and 740 nm excitation with respective power densities $\sim 9 \times 10^3$ W/cm^2 and $\sim 1.3 \times 10^2$ W/cm^2, at 3.8 K sample temperature. A very long period of anomalous Raman signal (probably coming from one or few molecules) is observable, while the PL continues to blink independently of that. (**c**) Intensity traces and (**d**) corresponding probability density functions (PDFs) for PL (orange) and Raman scattering (blue and purple for the peak at 803 nm, grey for the peak emerging temporarily at 811 nm). We do note a slightly increased blinking during, and persisting after, the anomalous Raman event, suggesting that the latter is caused by a metal restructuring that can also contribute to blinking. More research is needed to establish the relationship between anomalous Raman and blinking PL.

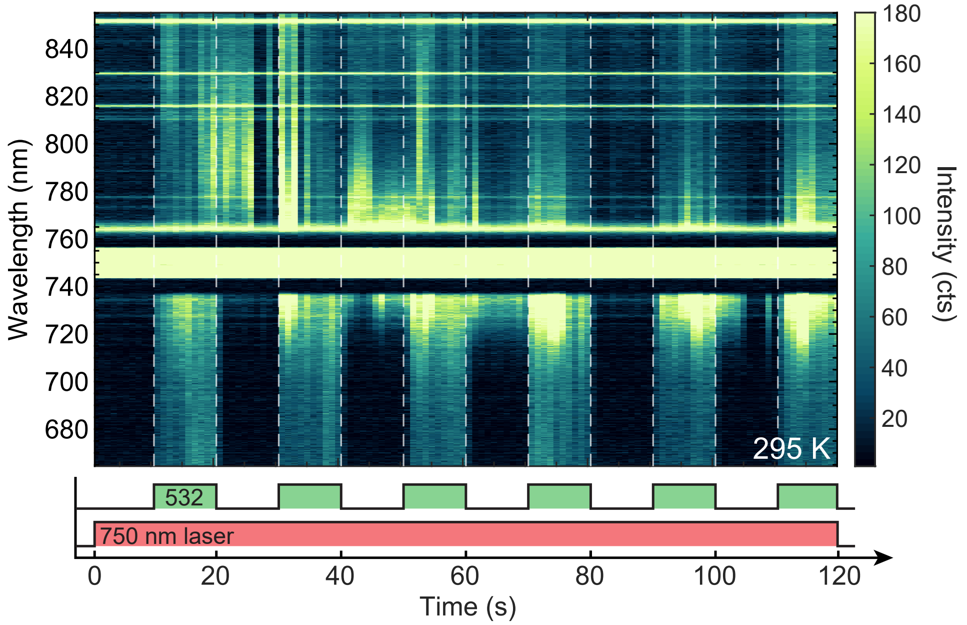

Supplementary Figure 15: Blinking activation by interband excitation. Two-color Raman+PL measurement with continuous excitation at 750 nm and intermittent excitation (periods of 10 s ON followed by 10 s OFF) at 532 nm (on sample No.2 in Supplementary Table 1). The experimental parameters are the same as in Fig. 3 in the main text. At the start of the experiment under 750 nm excitation alone the background emission is virtually absent, only the Raman signal is visible. After the 532 nm illumination is switched ON for 10 s and OFF again, however, we observe a persistently increased background emission under 750 nm excitation alone, suggesting that the blinking emission centers have been activated by the green laser. The result indicates that interband transitions and the generation of non-thermal photoexcited electrons play an important role in the activation of blinking. This observation is in line with the temperature-dependent measurements shown in the main text and heat simulations presented below, as it supports a non-thermal activation channel of blinking emitters.

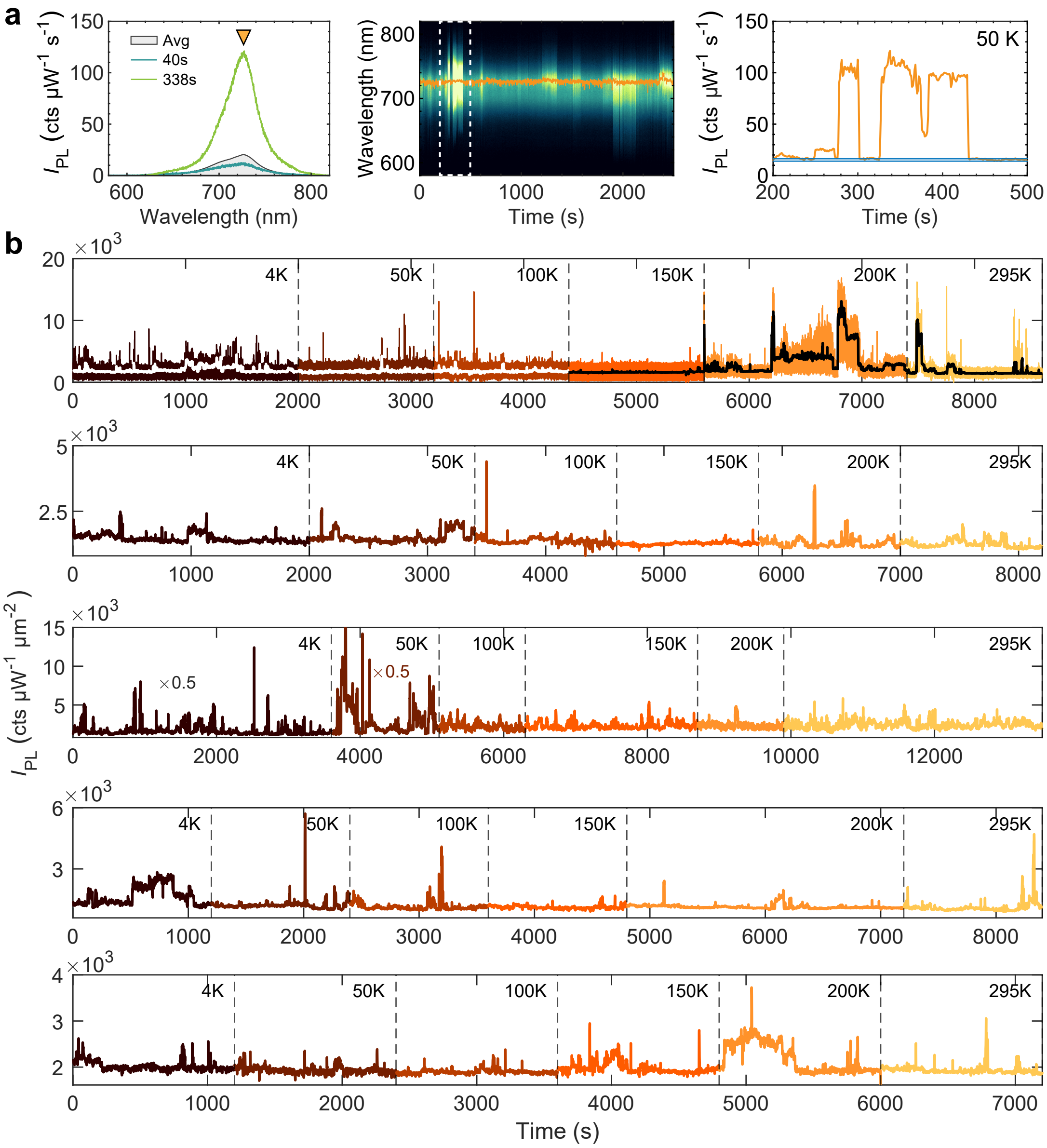

Supplementary Figure 16: Temperature-dependent PL measurements. (**a**) Time series of PL, selected PL spectra and peak intensity trace from a single nanojunction (on sample No. 7 in Supplementary Table 1) at 50 K by 532 nm excitation. Laser power, 32 µW, integration time, 1 s. (**b**) Time series of the PL peak intensity from different individual nanojunctions (from No. 19 in Supplementary Table 1) as a function of temperature measured by a single photon counting module. The first panel shows both 1 s and 1 ms binning time, while the traces in the remaining panel are binned to 1 s. No clear correlation between the blinking behavior and sample temperature can be found, with occasional periods of increased blinking occurring at different temperatures, case by case. This strongly favour a mechanism that is not thermally activated.

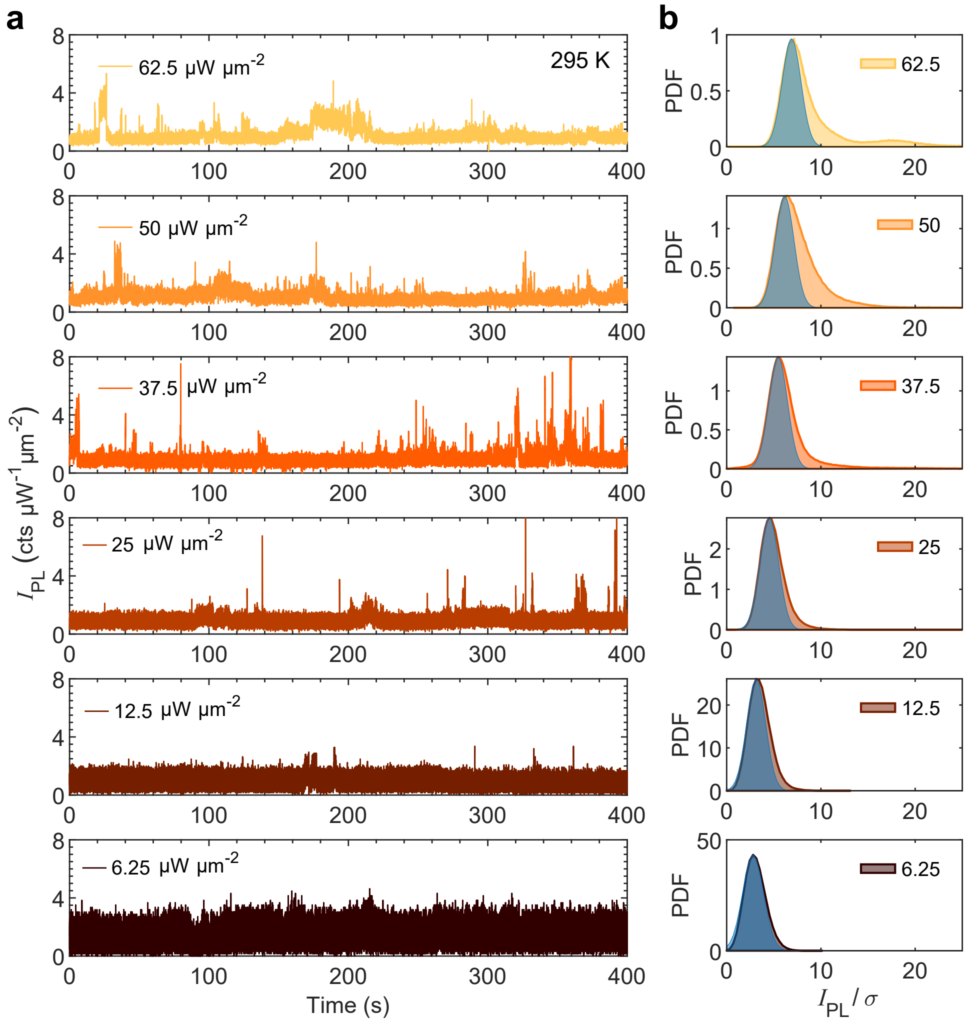

Supplementary Figure 17: Power-dependent PL measurements. Excitation power (at 532 nm) dependence of the PL intensity from a single nanojunctions (No. 7 in Supplementary Table 1) at room temperature. (**a**) PL intensity traces at different laser powers collected by a single photon counting module with 1 ms binning time. (**b**) Corresponding probability density functions (PDFs) of the traces compared with PDF of the corresponding intrinsic measurement noise (dark blue shade). The measured counts I_{PL} is normalized by the standard deviation σ to make the histograms comparable. Figure 5C in the main text is plotted by merging the six panels together.

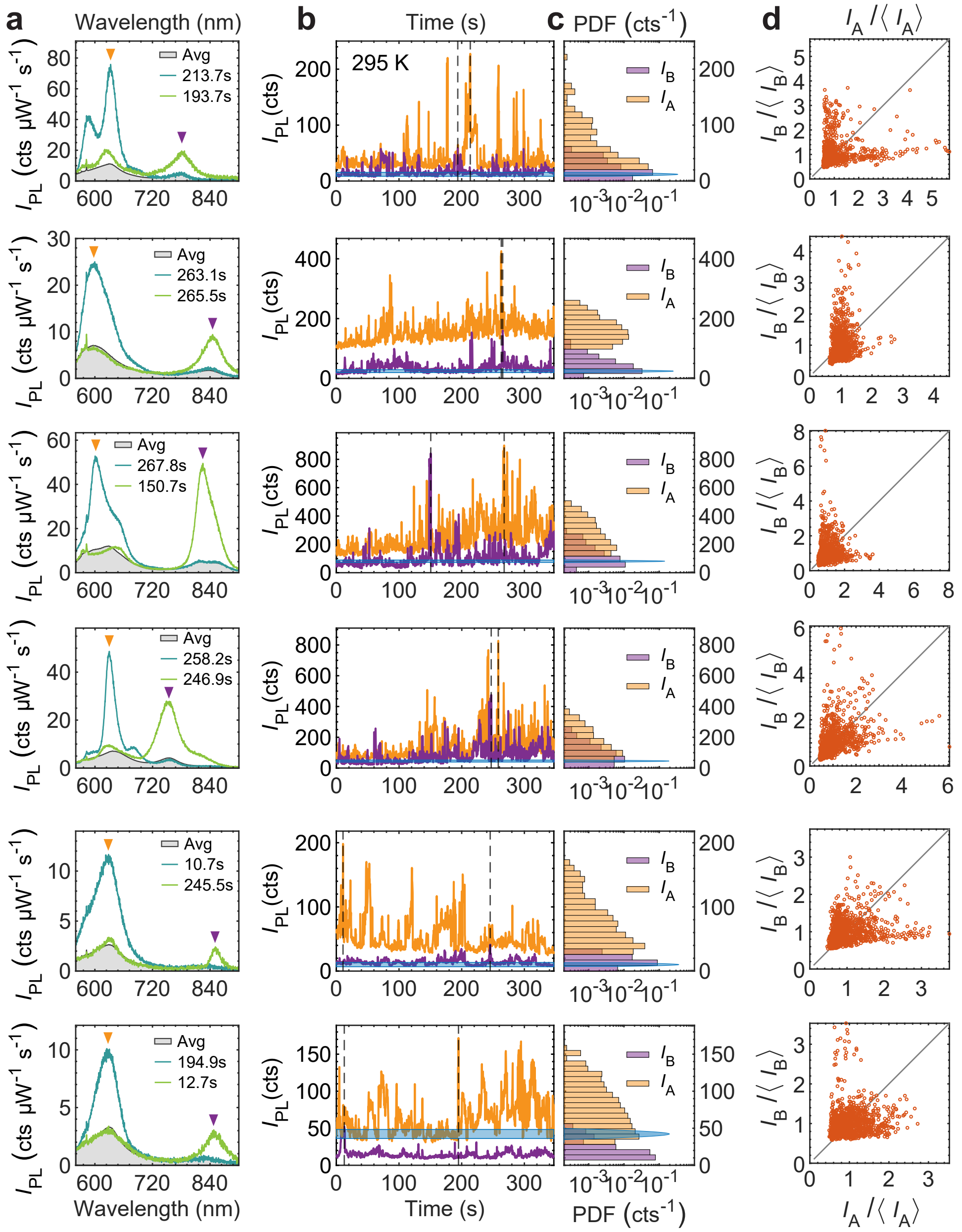

Supplementary Figure 18: Multi-peak analysis of PL blinking from additional examples. PL time series from 6 different individual nanojunctions under 532 nm excitation with 0.1s exposure time (from sample No. 3 in Supplementary Table 1). (**a**) Representative PL spectra of respective nanojunctions. (**b**) Intensity trace and (**c**) corresponding probability density functions of the PL peak A (<700 nm, orange) and B (>700 nm, purple). Shaded blue areas represent the noise associated with each of these measurements. (**d**) Correlation map between the intensity fluctuations of peaks A and B. No clear correlation between the PL of the different plasmonic modes can be evidenced.

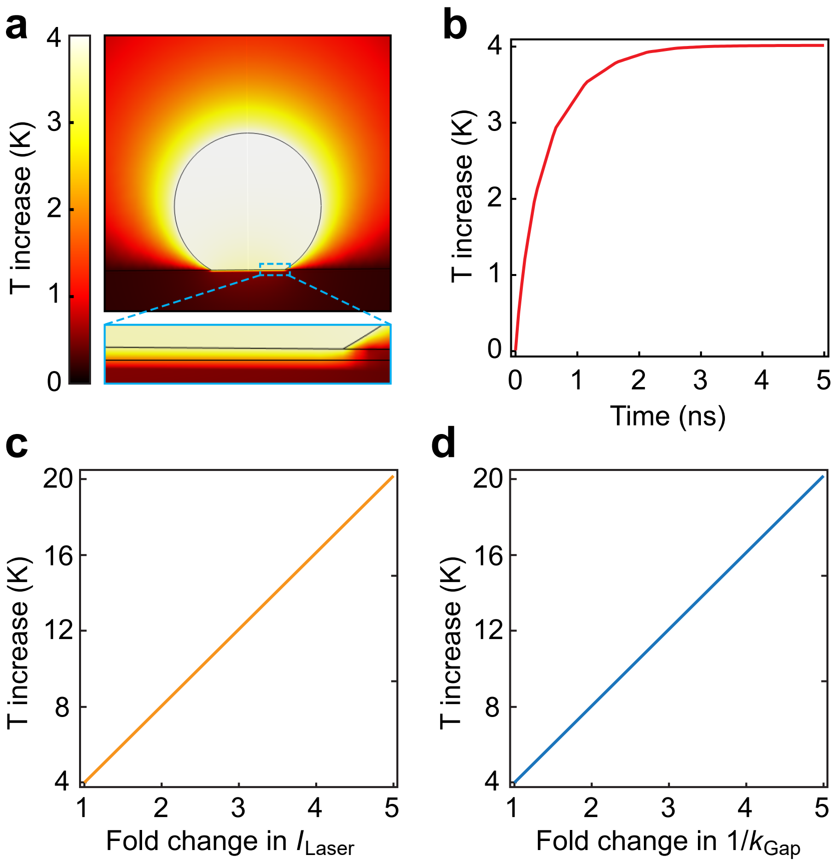

Supplementary Figure 19: Simulation of the temperature increase from a nanojunction by laser heating. (**a**) Temperature increase distribution with $\sim 3.5 \times 10^4$ W/cm^2 light excitation at 532 nm. (**b**) Time-dependent temperature increase, showing saturation of the temperature increase after a few nanoseconds. (**c**) The heat absorption and temperature increase linearly by increasing laser power I_{Laser}. (**d**) Temperature increase as a function of thermal conductivity in the gap k_{Gap}. The small temperature increase results from the high thermal conductivity of the gold substrate.

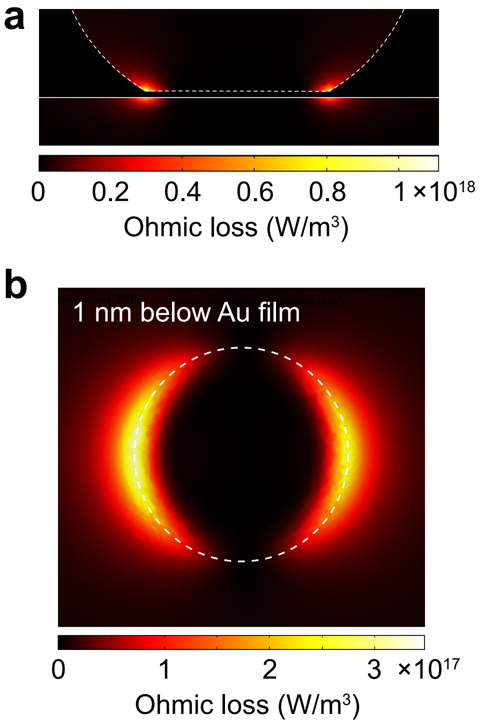

Supplementary Figure 20: Estimation of photon absorption rate by the nanojunction. Vertical cross-section (**a**) and in-plane (**b**, 1 nm below the Au film top surface) views of the simulated absorption distribution in a nanojunction illuminated with 3.5×10^4 W/cm^2 power density of monochromatic light at 532 nm (largest power used in the experiment). All parameters are the same as in Supplementary Fig. 19 and are described in the preceding text. The spatially integrated absorbed power from the gap region is $P_{\mathrm{Gap}} = 3.7 \times 10^{-6}$ W. We can translate this into the number of photons absorbed from the gap region within a duration T through $N_{\mathrm{Abs}} = P_{\mathrm{Gap}} T/\hbar\omega$, where $\hbar$ is the reduced Planck constant and ω is the laser frequency at 532 nm. The resulting N_{Abs} is about 10 photons per picosecond, where 1 ps is the timescale for energy relaxation through electron-phonon interaction. The 532 nm laser power used in our experiment to observe blinking is generally 10 times smaller, which gives one absorbed photon per picosecond, as quoted in the text.

Supplementary Note 1: A brief review of fluctuating SERS continuum from plasmonic hot-spots in comparison with PL blinking

In this section, we give a brief overview of previous observations of fluctuating emission from photo-excited plasmonic hot-spots, mainly in the context of surface-enhanced Raman scattering (SERS), and of the proposed mechanisms. We stress upfront that the vast majority of the literature on the topic is related to Raman signal fluctuations typically considered as a signature of single-molecule SERS (SM-SERS).[13–22] Overall, although a variety of models have been proposed to understand signal fluctuations in SM-SERS (and the origin of the gigantic signal enhancement), the underlying principles are still under debate due to the complex interactions between adsorbates and plasmonic hot-spots, and their evolution in the course of the experiment. The samples used in previous research on SM-SERS were most frequently fabricated by the mixture of salt-aggregated Ag nanoparticles and a very low concentration of analyte molecules (rhodamine 6G, crystal violet, biomolecules, etc.)[13–16] Without accurate control, it was found that the analyte randomly diffuses in and out of the hot-spot region and that the molecular orientation varies,[23,24] leading to fluctuations of the Raman scattering spectrum and intensity.[21,25–29] Interestingly, the broad emission underlying the Raman peaks (called 'SERS continuum'), which is absent from powders and pure molecule ensembles, was found to fluctuate together with the Raman signal in numerous previous reports.[15,18,27,30–36] While much research crystallized on elucidating the origin of the 'SERS continuum', no single mechanism was yet demonstrated to account for all experimental data.

On the contrary, in plasmonic nanojunctions with well-controlled geometry used in our work, metal luminescence blinking is not accompanied by measurable Raman signal fluctuations. Therefore, the above-mentioned phenomenology cannot be mapped onto our observations. On the other hand, owing to the similarity in the phenomenon, the 'SERS continuum' fluctuations – reported mostly from silver particles – could be intuitively considered as sharing the same mechanisms as the PL blinking reported here in gold nanojunctions. Nevertheless, these two phenomena actually feature significant differences. To further clarify their relationship and differences, we review below the main hypotheses for the origin of the 'SERS continuum' fluctuations, and assess them against our observation of PL blinking.

Main fluctuation mechanisms proposed to date

One possible origin for the intensity fluctuation of the 'SERS continuum' is a variation of the local field enhancement, which can be caused either by a global effect involving the entire plasmonic structures[24,29,37] (e.g., change of the gap size) or a local effect involving adatom diffusion dynamics on the metal surface.[38,39] The latter was proposed to cause atomic-scale confinement of light and activate new Raman modes in the surrounding molecules due to the large field gradient (a phenomenon that we also report in Supplementary Fig. 14). As our simultaneous PL+Raman and PL+DF measurements demonstrate, such a mechanism cannot explain the pronounced intrinsic PL blinking that we report here. In particular, the results of Fig. 2

in the main text suggests the independent generation of PL emission coupled to different plasmonic modes, which together with the PL+DF measurements (Fig. 4 in the main text) exclude any mechanism that would be linked with the change of the bulk plasmon frequency, as proposed in.[40]

A second possible fluctuation mechanism is based on dynamical charge transfer between adsorbate and metal, which is sometimes referred to as the chemical enhancement factor in SERS.[20,22,26,27,29,35,41] In this model, spectral fluctuations are caused by chemical adsorption or desorption of the molecule, or by its thermally activated atomic scale movement. It was also proposed that such change of adsorbate-metal interaction can give rise to the fluctuation of the SERS continuum. This model relates the SERS continuum to an electronic Raman process that is enhanced by the relaxation of momentum conservation when a chemisorbed molecule acts as a localised 'defect' on the metal surface.[23,32,42–44] In light of our data, this mechanism could explain some blinking events in which a plasmonic mode becomes momentarily brighter while conserving its line shape and peak wavelength. However, by comparing the various types of samples we synthesized, we see that PL blinking also happens in the plasmonic nanojunctions without molecular chemisorption (e.g. dielectric and citrate spacers from samples No. 17 and 19 in Supplementary Table 1). As a result, we conclude that if an electronic Raman process is at play during some blinking periods, then it can also be induced by intrinsic defects in the metal surface layers, and not only by adsorbed moieties.

Recently, the variation of bulk plasma frequency induced by local defects on the metal interface was proposed as a mechanism to explain the fluctuating 'SERS continuum' under stable Raman signal in plasmonic hot-spots.[40] Although this phenomenon seems at first similar to our results from the two-color PL+Raman measurement (Fig. 3 in the main text), the 'SERS continuum' fluctuations in[40] are predicted to be correlated with a pronounced shift of the entire plasmonic resonance spectrum. It is at odds with our observations of stable DF scattering spectrum during blinking. On the other hand, the authors of[40] explain the 'SERS continuum' as electronic Raman scattering rather than electron-hole recombination process, which can apply to measurements under near-infrared excitation (Fig. S10c and S10d) but does not represent the dominant interband transition processes at play under green excitation.

Another possible source of spectral fluctuation is the contamination of amorphous carbon around the plasmonic hot-spots, which generally comes from the damage of carbon-based molecules under laser irradiation or heating from analyte, impurities from the solution, or even from the air (e.g., CO).[19,20,25,37,43,45–49] Amorphous carbon shows quite broad Raman bands with the strongest two peaks around 1300 cm^{-1} and 1600 cm^{-1} and their overtone modes around 3000 cm^{-1}, which are also recognised as a type of SERS continuum.[25,50,51] In our experiment, the stable Raman signal with low background under near-infrared light excitation (Fig. 3 in the main text) suggests that carbon contamination does not have any significant contribution to the PL blinking. This conclusion is supported by the high sensitivity of blinking behavior on spacer material, even though all samples are expected to be equally contaminated by amorphous carbon since they are prepared and studied in similar conditions. If PL blinking

were related to carbon contamination, we believe that the prominence and characteristics of PL blinking would be the same for all samples, which is clearly disproved by our PL measurements (Supplementary Fig. 10 and 11).

To place our work into context, it should be emphasized that most SERS blinking observations were reported from Ag systems, which show pronounced chemical reactivity,[30,33,43,52–57] in contrast to gold which is a rather inert substrate at ambient conditions. Indeed, luminescent Ag adatoms can be photochemically generated from a silver oxide system under laser irradiation.[33,43,52,53] Combining this effect with oxidation from Ag to Ag oxide in air, a reversible photochemical reaction loop can be realised, resulting in luminescence blinking even from a bare Ag system without any Raman probe.[33,54–56] In parallel, Ag can also exhibit a strong interaction with CO from the environment; the Raman signal of carbon contaminants can even be found from fresh Ag films deposited under high vacuum conditions.[47,50] All these effects make the blinking phenomenon in Ag systems easier to observe but more complicated to analyze compared to Au systems, which is why we focused our main study on gold. There is rare literature reporting luminescence fluctuations from Au systems, but existing reports either lack control over the structures and corresponding local fields[58] or independent characterisation of the plasmonic response and possible changes in the local fields.[59]

Clarifying the underlying mechanisms relies on developing a stable plasmonic platform with much better control of morphology compared to previous systems, as demonstrated in our nanoparticle-on-mirror systems. In contrast with previous reports where the continuum emission fluctuated together with the Raman signal[15,18,27,30–36] (or without discrimination between them), our observation of PL blinking without Raman blinking clearly evidences that other changes are occurring within the metal instead of inside the gap. This excludes mechanisms involving molecule diffusion or local field variation (such as ‘picocavities’). More generally, unlike in Ag systems, it is improbable that any photochemical reactions take place between gold and the immediate environment.[60–62] Luminescence blinking is also unlikely to arise from the ‘off-resonant’ molecules (i.e., do not absorb nor luminescence at the relevant wavelengths) used in our plasmonic nanojunctions.

Supplementary Note 2: Comparison of PL blinking from different types of nanojunctions

To test the generality of the blinking phenomenon, and to investigate the impact of nanojunction composition on blinking, we fabricated and characterised many different types of nanojunctions (see Supplementary Table 1), and some measurement examples are presented in Supplementary Fig. 10 and Supplementary Fig. 11. As explained above, we systematically changed the substrate type, the spacer layer, and the nanoparticle material and shape, while maintaining similar plasmonic resonance frequencies and mode volumes. A general conclusion can be drawn from these measurements: the occurrence frequency, duration and strength of the PL fluctuations are governed mostly by the metal surface chemistry; on the contrary, PL blinking seems to weakly depend on the degree of crystallinity of the substrate, or on the composition and shape of the nanoparticle forming the junction. This highlights the key role of photo-induced restructuring of the metal surface layer.

Fluctuations are found to be more pronounced in the nanojunctions with molecules linked by thiol groups to the Au film surface (Supplementary Fig. 10a, 11a, 11b, 11d and 11f), while the structures with an oxide layer separating the Au film from the molecules tend to yield more stable emission, with only short bursts lasting less than one second (Supplementary Fig. 10b and 10c). This suggests the significantly larger contribution from Au film to the blinking emission than from the nanoparticles.

When using a single layer MoS_2 as a robust crystalline spacer, which strongly binds to both the Au nanoparticle and film by sulfur atoms (Supplementary Fig. 10e), PL fluctuations are not as pronounced as with Au-molecule-Au configurations. This highlights the role of molecular species in destabilising the metal surface layer and promoting light-induced cluster formation.

Silver nanojunctions blink much more strongly than Au nanojunctions, even though the Ag film is covered by a compact oxide layer, and only native ligand molecules may remain (Supplementary Fig. 10f). This may be because the chemical reactivity of Ag and the field enhancement are higher than those of Au, which probably boost the formation of atomic clusters responsible for blinking emission. Remarkably, the emission enhancement reaches almost 6 orders of magnitude compared to the substrate emission, which corresponds to approximately 8 to 9 orders of magnitude enhancement factor when accounting for the size of the nanojunction. The resulting PL quantum yield seems on par with that of semiconducting emitters (but we did not perform a quantitative quantum yield measurement).

In order to clarify the relationship between PL blinking and the existence of a nano-gap yielding extreme field confinement, we study the PL of an individual 150-nm-diameter Au nanoparticle on a 100-nm-thick SiO_2 layer over a Si substrate (Supplementary Fig. 12). As shown in Supplementary Fig. 12d and 12e, both simulated and measured scattering spectra show a broad plasmonic resonance covering 600-700 nm, which can be identified as a dipole mode based on the charge distribution (inset in Supplementary Fig. 12d). Under s-polarised excitation, the dipole mode is equivalent to the transverse mode of the nanojunction. The dipole mode shows a red-shift under p-polarised excitation (Supplementary Fig. 12d and 12e) because

more charge is localised in the bottom facet causing stronger coupling with the substrate. An additional peak near the excitation wavelength (beneath the Si Raman peaks) can be found in the PL spectrum (Supplementary Fig. 12e), which originates from the edge of interband transitions in gold.[11,63,64]

An example of PL time-series measurement is shown in Supplementary Fig. 12e and 12f. While the PL spectra of the individual nanoparticle are generally stable compared to the nanojunctions, we do observe rare and weak blinking events (Supplementary Fig. 12g). Note that citrate molecules are loosely covered on the nanoparticle, which is in line with our proposed mechanism that the presence of molecules at the surface plays an important role in facilitating adatom migration and the generation of atomic defects. This very low blinking occurrence may also explain why PL blinking effect was not reported from most-studied individual metal nanoparticles (e.g. nanorods).[11,65] From this comparison, we conclude that the much higher field enhancement provided by the nanoparticle-on-mirror (at least one order of magnitude larger than at the surface of isolated particles) is essential in triggering the blinking mechanism.

Supplementary References

1. Chen, W., Zhang, S., Deng, Q. & Xu, H. Probing of sub-picometer vertical differential resolutions using cavity plasmons. *Nat. Commun.* **9**, 801 (2018).

2. Castellanos-Gomez, A. *et al.* Deterministic transfer of two-dimensional materials by all-dry viscoelastic stamping. *2D Mater.* **1**, 011002 (2014).

3. Zhou, S. *et al.* Facile Synthesis of Silver Nanocubes with Sharp Corners and Edges in an Aqueous Solution. *ACS Nano* **10**, 9861–9870 (2016).

4. Chen, W. *et al.* Probing the limits of plasmonic enhancement using a two-dimensional atomic crystal probe. *Light Sci. Appl.* **7**, 56 (2018).

5. Sigle, D. O. *et al.* Monitoring Morphological Changes in 2D Monolayer Semiconductors Using Atom-Thick Plasmonic Nanocavities. *ACS Nano* **9**, 825–830 (2015).

6. Johnson, P. B. & Christy, R. W. Optical Constants of the Noble Metals. *Phys. Rev. B* **6**, 4370–4379 (1972).

7. Young, H. D. *University Physics.* (Addison-Wesley Pub. Co., Reading, Mass, 1992), 8th ed. edn.

8. Cappella, A. *et al.* High Temperature Thermal Conductivity of Amorphous Al $_2$ O $_3$ Thin Films Grown by Low Temperature ALD: High Temperature Thermal Conductivity of α-Al $_2$ O $_3$ by ALD. *Adv. Eng. Mater.* **15**, 1046–1050 (2013).

9. Homola, J. & Piliarik, M. Surface Plasmon Resonance (SPR) Sensors. In Homola, J. (ed.) *Surface Plasmon Resonance Based Sensors*, vol. 4, 45–67 (Springer Berlin Heidelberg, Berlin, Heidelberg, 2006).

10. Lumdee, C., Yun, B. & Kik, P. G. Gap-Plasmon Enhanced Gold Nanoparticle Photoluminescence. *ACS Photonics* **1**, 1224–1230 (2014).

11. Cai, Y.-Y. *et al.* Photoluminescence of Gold Nanorods: Purcell Effect Enhanced Emission from Hot Carriers. *ACS Nano* **12**, 976–985 (2018).

12. McDonnell, S., Addou, R., Buie, C., Wallace, R. M. & Hinkle, C. L. Defect-Dominated Doping and Contact Resistance in MoS $_2$. *ACS Nano* **8**, 2880–2888 (2014).

13. Nie, S. Probing Single Molecules and Single Nanoparticles by Surface-Enhanced Raman Scattering. *Science* **275**, 1102–1106 (1997).

14. Kneipp, K. *et al.* Single Molecule Detection Using Surface-Enhanced Raman Scattering (SERS). *Phys. Rev. Lett.* **78**, 1667–1670 (1997).

15. Michaels, A. M., Nirmal, M. & Brus, L. E. Surface Enhanced Raman Spectroscopy of Individual Rhodamine 6G Molecules on Large Ag Nanocrystals. *J. Am. Chem. Soc.* **121**, 9932–9939 (1999).

16. Xu, H., Bjerneld, E. J., Käll, M. & Börjesson, L. Spectroscopy of Single Hemoglobin Molecules by Surface Enhanced Raman Scattering. *Phys. Rev. Lett.* **83**, 4357–4360 (1999).

17. Bjerneld, E. J., Johansson, P. & Käll, M. Single Molecule Vibrational Fine-structure of Tyrosine Adsorbed on Ag Nano-Crystals. *Single Molecules* **1**, 239–248 (2000).

18. Michaels, A. M., Jiang & Brus, L. Ag Nanocrystal Junctions as the Site for Surface-Enhanced Raman Scattering of Single Rhodamine 6G Molecules. *J. Phys. Chem. B* **104**, 11965–11971 (2000).

19. Otto, A. What is observed in single molecule SERS, and why? *J. Raman Spectrosc.* **33**, 593–598 (2002).

20. Kudelski, A. Some aspects of SERS temporal fluctuations: Analysis of the most intense spectra of hydrogenated amorphous carbon deposited on silver. *J. Raman Spectrosc.* **38**, 1494–1499 (2007).

21. Stranahan, S. M. & Willets, K. A. Super-resolution optical imaging of single-molecule SERS hot spots. *Nano Lett.* **10**, 3777–84 (2010).

22. Lombardi, J. R., Birke, R. L. & Haran, G. Single Molecule SERS Spectral Blinking and Vibronic Coupling. *J. Phys. Chem. C* **115**, 4540–4545 (2011).

23. Jiang, Bosnick, K., Maillard, M. & Brus, L. Single Molecule Raman Spectroscopy at the Junctions of Large Ag Nanocrystals. *J. Phys. Chem. B* **107**, 9964–9972 (2003).

24. Maruyama, Y., Ishikawa, M. & Futamata, M. Thermal Activation of Blinking in SERS Signal. *J. Phys. Chem. B* **108**, 673–678 (2004).

25. Moyer, P. J. *et al.* Surface-Enhanced Raman Scattering Spectroscopy of Single Carbon Domains on Individual Ag Nanoparticles on a 25 ms Time Scale. *J. Am. Chem. Soc.* **122**, 5409–5410 (2000).

26. Otto, A. Theory of First Layer and Single Molecule Surface Enhanced Raman Scattering (SERS). *Phys. Status Solidi* **188**, 1455–1470 (2001).

27. Weiss, A. & Haran, G. Time-Dependent Single-Molecule Raman Scattering as a Probe of Surface Dynamics. *J. Phys. Chem. B* **105**, 12348–12354 (2001).

28. Futamata, M., Maruyama, Y. & Ishikawa, M. Microscopic morphology and SERS activity of Ag colloidal particles. *Vib. Spectrosc.* **30**, 17–23 (2002).

29. Emory, S. R., Jensen, R. A., Wenda, T., Han, M. & Nie, S. Re-examining the origins of spectral blinking in single-molecule and single-nanoparticleSERS. *Faraday Discuss.* **132**, 249–259 (2006).

30. Mihalcea, C., Büchel, D., Atoda, N. & Tominaga, J. Intrinsic Fluorescence and Quenching Effects in Photoactivated Reactively Sputtered Silver Oxide Layers. *J. Am. Chem. Soc.* **123**, 7172–7173 (2001).

31. Meixner, A. J., Vosgröne, T. & Sackrow, M. Nanoscale surface-enhanced resonance Raman scattering spectroscopy of single molecules on isolated silver clusters. *J. Lumin.* **94-95**, 147–152 (2001).

32. Bosnick, K. A., Jiang & Brus, L. E. Fluctuations and Local Symmetry in Single-Molecule Rhodamine 6G Raman Scattering on Silver Nanocrystal Aggregates. *J. Phys. Chem. B* **106**, 8096–8099 (2002).

33. Andersen, P. C., Jacobson, M. L. & Rowlen, K. L. Flashy Silver Nanoparticles. *J. Phys. Chem. B* **108**, 2148–2153 (2004).

34. Itoh, T. *et al.* Elucidation of Interaction between Metal-Free Tetraphenylporphine and Surface Ag Atoms through Temporal Fluctuation of Surface-Enhanced Resonance Raman Scattering and Background-Light Emission. *J. Phys. Chem. B* **110**, 9579–9585 (2006).

35. Bizzarri, A. R. & Cannistraro, S. Statistical analysis of intensity fluctuations in single molecule SERS spectra. *Phys. Chem. Chem. Phys.* **9**, 5315–5319 (2007).

36. Weber, M. L., Litz, J. P., Masiello, D. J. & Willets, K. A. Super-Resolution Imaging Reveals a Difference between SERS and Luminescence Centroids. *ACS Nano* **6**, 1839–1848 (2012).

37. Kudelski, A. Fluctuations of Raman spectra of hydrogenated amorphous carbon deposited on electrochemically-roughened silver. *Chem. Phys. Lett.* **427**, 206–209 (2006).

38. Benz, F. *et al.* Single-molecule optomechanics in “picocavities”. *Science* **354**, 726–729 (2016).

39. Shin, H.-H. *et al.* Frequency-Domain Proof of the Existence of Atomic-Scale SERS Hot-Spots. *Nano Lett.* **18**, 262–271 (2018).

40. Carnegie, C. *et al.* Flickering nanometre-scale disorder in a crystal lattice tracked by plasmonic flare light emission. *Nat. Commun.* **11**, 1–9 (2020).

41. Moskovits, M. Surface-enhanced spectroscopy. *Rev. Mod. Phys.* **57**, 783–826 (1985).

42. Gass, A. N., Kapusta, O. I., Klimin, S. A. & Mal’shukov, A. G. The nature of the inelastic background in surface enhanced raman scattering spectra of coldly-deposited silver films. The role of active sites. *Solid State Commun.* **71**, 749–753 (1989).

43. Monti, O. L. A., Fourkas, J. T. & Nesbitt, D. J. Diffraction-Limited Photogeneration and Characterization of Silver Nanoparticles. *J. Phys. Chem. B* **108**, 1604–1612 (2004).

44. Moore, A. A., Jacobson, M. L., Belabas, N., Rowlen, K. L. & Jonas, D. M. 2D Correlation Analysis of the Continuum in Single Molecule Surface Enhanced Raman Spectroscopy. *J. Am. Chem. Soc.* **127**, 7292–7293 (2005).

45. Tsang, J. C., Demuth, J. E., Sanda, P. N. & Kirtley, J. R. Enhanced raman scattering from carbon layers on silver. *Chem. Phys. Lett.* **76**, 54–57 (1980).

46. Kudelski, A. & Pettinger, B. SERS on carbon chain segments: Monitoring locally surface chemistry. *Chem. Phys. Lett.* **321**, 356–362 (2000).

47. Kudelski, A. & Pettinger, B. Fluctuations of surface-enhanced Raman spectra of CO adsorbed on gold substrates. *Chem. Phys. Lett.* **383**, 76–79 (2004).

48. Lucotti, A. *et al.* Raman and SERS investigation of isolated sp carbon chains. *Chem. Phys. Lett.* **417**, 78–82 (2006).

49. Chaigneau, M., Picardi, G. & Ossikovski, R. Tip enhanced Raman spectroscopy evidence for amorphous carbon contamination on gold surfaces. *Surf. Sci.* **604**, 701–705 (2010).

50. Mrozek, I., Pettenkofer, C. & Otto, A. Raman spectroscopy of carbon monoxide adsorbed on silver island films. *Surf. Sci.* **238**, 192–198 (1990).

51. Robertson, J. Diamond-like amorphous carbon. *Mater. Sci. Eng. R Rep.* **37**, 129–281 (2002).

52. Peyser, L. A., Vinson, A. E., Bartko, A. P. & Dickson, R. M. Photoactivated Fluorescence from Individual Silver Nanoclusters. *Science* **291**, 103–106 (2001).

53. Peyser, L. A., Lee, T.-H. & Dickson, R. M. Mechanism of Ag_n Nanocluster Photoproduction from Silver Oxide Films. *J. Phys. Chem. B* **106**, 7725–7728 (2002).

54. Jacobson, M. L. & Rowlen, K. L. Photo-dynamics on thin silver films. *Chem. Phys. Lett.* **401**, 52–57 (2005).

55. Jacobson, M. L. & Rowlen, K. L. The Role of O2 in SERS-Active Thin Metal Film Photodynamics. *J. Phys. Chem. B* **110**, 19491–19496 (2006).

56. Wu, X. & Yeow, E. K. L. Fluorescence blinking dynamics of silver nanoparticle and silver nanorod films. *Nanotechnology* **19**, 035706 (2008).

57. Borys, N. J. & Lupton, J. M. Surface-Enhanced Light Emission from Single Hot Spots in Tollens Reaction Silver Nanoparticle Films: Linear versus Nonlinear Optical Excitation. *J. Phys. Chem. C* **115**, 13645–13659 (2011).

58. Geddes, C. D., Parfenov, A., Gryczynski, I. & Lakowicz, J. R. Luminescent blinking of gold nanoparticles. *Chem. Phys. Lett.* **380**, 269–272 (2003).

59. Li, T. *et al.* Revealing the Mechanism of Photoluminescence from Single Gold Nanospheres by Defocused Imaging. *ACS Photonics* **4**, 2003–2010 (2017).

60. Boyen, H.-G. Oxidation-Resistant Gold-55 Clusters. *Science* **297**, 1533–1536 (2002).

61. Tsai, H. *et al.* Instability of gold oxide Au2O3. *Surf. Sci.* **537**, L447–L450 (2003).

62. Ono, L. K. & Roldan Cuenya, B. Formation and Thermal Stability of Au_2O_3 on Gold Nanoparticles: Size and Support Effects. *J. Phys. Chem. C* **112**, 4676–4686 (2008).

63. Yorulmaz, M., Khatua, S., Zijlstra, P., Gaiduk, A. & Orrit, M. Luminescence Quantum Yield of Single Gold Nanorods. *Nano Lett.* **12**, 4385–4391 (2012).

64. Fröhlich, T., Schönenberger, C. & Calame, M. Additional peak appearing in the one-photon luminescence of single gold nanorods. *Opt. Lett.* **41**, 1325–1328 (2016).

65. Fang, Y. *et al.* Plasmon Emission Quantum Yield of Single Gold Nanorods as a Function of Aspect Ratio. *ACS Nano* **6**, 7177–7184 (2012).
