# Supplementary material for: Intrinsic luminescence blinking from plasmonic nanojunctions
Source: Nat Commun. 2021 May 21;12:2731. doi: 10.1038/s41467-021-22679-y (PMC8139969; doi:10.1038/s41467-021-22679-y)
Supplement: Supplementary file 3 — Description of Additional Supplementary Files [file 41467_2021_22679_MOESM3_ESM.docx]

**Description of Additional Supplementary Files**

**Supplementary Movie 1:** The video for the PL spectra time trace of a nanojunction, from the same data shown in Fig. 1d in the main text.

**Supplementary Movie 2:** A real-time image of simultaneous PL and DF measurement. It shows stable DF while PL is blinking, similar to another measurement shown in Fig. 4a in the main text.
